# Protocol for the CONNECT Study: A National Database and Prospective Follow-Up Study of Forensic Mental Health Patients in Germany

**DOI:** 10.3389/fpsyt.2022.827272

**Published:** 2022-04-25

**Authors:** Jack Tomlin, Peggy Walde, Birgit Völlm

**Affiliations:** ^1^School of Law and Criminology, University of Greenwich, London, United Kingdom; ^2^Department for Forensic Psychiatry, Rostock University Medical Center, Rostock, Germany

**Keywords:** forensic psychiatry, prospective, reoffending, treatment outcomes, database, survey, Germany

## Abstract

In Germany, the most frequently used legal section to order forensic mental health treatment is § 63 of the Penal Code (Strafgesetzbuch; StGB). This disposition is primarily aimed at individuals with major mental illnesses who are not fully responsible for a criminal act they committed. Despite evaluation and follow-up studies being conducted within individual hospitals or federal states we lack key epidemiological data on this patient group across the whole country. The present study aims to fill this gap by conducting an annual survey of all eligible forensic mental health hospitals to develop a database of basic clinical, legal and demographic data. Staff at participating hospitals will complete an online survey answering questions about individual patients using routinely collected hospital records. Over the duration of the study, eight-and-a-half years, we aim to collect data on approximately *N* = 6,450 patients. Alongside important clinical data, we will use official reconviction data at 3- and 6-year follow-ups to investigate the number and types of crimes committed by discharged patients. We aim to extend the scientific literature on factors associated with reconviction in the Risk-Needs-Responsivity model by also measuring the extent to which treatment engagement and programme completion during care predicts reconviction. This study protocol describes the background and theoretical framework for this study, its methods of data collection and analysis, and steps taken to ensure compliance with ethical and data protection principles.

## Introduction

### Forensic Mental Health Services in Germany

Forensic mental health services provide care to individuals who have committed a criminal offense, the commission of which is related to a mental disorder. In Germany, a court may order mental health treatment for individuals found not criminally responsible or not fully criminally responsible for the act under sections §§20 or 21 of the Penal Code (StGB), respectively, and who pose a risk of harm. Orders for forensic treatment are provided for in §§63 and 64 StGB, which, respectively, allow for the indefinite detention of individuals typically diagnosed with a major mental illness or time-limited detention of patients with a substance use disorder. Under both dispositions, there must exist a risk of future harm committed by the patient, and for those detained under §64 StGB treatment is expected to carry mental health and criminogenic benefit.

The two main aims of forensic care for this group are to reduce the risk of future harms, including reconviction and violence, and to treat patients' mental disorders, thereby facilitating recovery and reintegration into society (1). Patients receiving care under §63 StGB are most commonly diagnosed in accordance with the International Classification of Diseases (ICD-10 or 11) with severe mental disorders including psychosis, personality disorder, intellectual disability and substance use disorder (2, 3). Treatment is multimodal involving a range of interventions targeting mental health, social functioning and criminogenic factors (3). Success is indicated by symptom alleviation, insight into illness and offending, reduction of risk, and progression toward lower levels of restriction, including discharge from services (2). Treatment under §63 StGB is provided in secure inpatient psychiatric hospitals.^1^ Treatment in the community or other settings is ordered under different legal sections.

According to point prevalence data collected by the Federal Office of Statistics there were at least 5,926 § 63 StGB patients on the 31.03.2019, with 1,137 admitted across the year 2018 (data were not collected for four of the 16 federal states in Germany that represent 13% of the total general population) (4). Most patients are men [women are 9% (4)]. Average inpatient treatment duration is ~8 years (5). Most §63 StGB patients have experienced difficult upbringings and have complex social and health needs, often having been admitted to psychiatric care or imprisoned previously, not unlike forensic patients in other countries (6).

### Research Problem: A Lack of Robust National Data

We lack robust data on reconviction and treatment outcomes for forensic patients across the country. Whilst some organizations do collect descriptive patient data across multiple states, no effort systematically records clinical, demographic and legal data for all patients and follows these patients over time. There are several reasons why routinely collected, valid and reliable data are needed for this group. First, an epidemiological overview is needed to assist state and federal governments planning service provision. Second, we are currently unable to track how social developments such as changes in demography and the law are reflected in this group. Third, forensic services are expensive: a 2007 study concluded that the average yearly cost for a patient in Mecklenburg-Western Pomerania was between 82,198 € and 92,923 € (7). Discerning treatment methods that are associated with better patient engagement in care can improve efficiency/reduce costs. Fourth, forensic treatment is provided in highly restrictive hospitals for indefinite periods of time. There is a human rights imperative to ensure evidence-based treatment and improve outcomes for patients and marginalized social groups (8). Finally, a better understanding of what works to reduce reoffending helps us to reduce risk and protect the public. One solution to this is the development of a longitudinal, national survey and database (9).

One of the most extensive studies of §63 StGB patients reported data on 80% of all patients but was published 33 years ago (10). The Center for Forensic Psychiatry in Reichenau maintains a local database of all forensic patients receiving care in the state of Baden-Württemberg. The Institute for Quality Management in Forensic Psychiatry in Bavaria (IFQM) collects similar data on a routine basis from 12 hospitals in that state (11). The private consulting company CEUS-Consulting has been commissioned by 14 federal states to conduct a survey (Massregelvollzug-Kerndatensatz) on forensic patients. However, data are not publicly available, the dataset excludes Bavaria and Baden-Württemberg (30% of the forensic population), any analyses that have been conducted have not been published in the international literature, and all data are at aggregate level and thus not amenable to individual statistical analysis or follow-up (12).

The above demonstrates that the clinical infrastructure and need for routinely collected data are present in forensic services. This project aims to fill this gap by annually collecting data on all §63 StGB patients from all eligible forensic hospitals in Germany over an 8.5-year period (102 months). In doing so, we also respond to a call from the Directorate General for Health and Consumers of the European Union to develop mental health information systems (13).

### Theoretical Framework

One of the most dominant frameworks in offender rehabilitation is the Risk-Need-Responsivity (RNR) model (14, 15). The present study aims to expand the theoretical content of the RNR model by investigating the relevance of “treatment engagement” variables in predicting risk of reconviction.

The RNR model is a practical framework for directing interventions to offenders at highest risk of reoffending (14). It proposes that interventions should target individuals' criminogenic needs and do so in an individualized and responsive way. Criminogenic needs (also called risk factors) are individual-level static and dynamic factors associated with reoffending. Andrews and Bonta have identified eight factors (the Central Eight) most associated with reoffending: (1) criminal history, (2) procriminal companions, (3) procriminal attitudes and cognitions, (4) antisocial personality patterns, (5) lack of education/employment, (6) poor or procriminal family/marital relationships, (7) substance abuse, and (8) lack of prosocial leisure/recreation (14). Services should be targeted at individuals who present with a variety and intensity of these risk factors. These services should be tailored (responsive) to that individual's needs and strengths; interventions that are responsive to individual needs are more effective at reducing reoffending (14).

An indicator of the suitability and responsiveness of an intervention is the extent to which a patient engages in treatment (16). It follows that treatment engagement and programme completion might be predictors of reoffending. However, few studies have tested the link between treatment engagement and reoffending in the forensic mental health population. Those that have investigated this found mixed results. One study reported that treatment engagement was associated with higher serious reoffending rates in sexual offenders (17), whilst another found a negative relationship in individuals after leaving treatment for substance misuse (18). Our understanding of the link between treatment engagement (and other dynamic treatment-related factors) and reoffending is underdeveloped. In this study, we will investigate whether treatment engagement and programme completion variables add significantly to models that predict reconviction alongside well-established risk factors identified in the RNR framework (e.g., sex, age, number of past criminal convictions). We will investigate this by asking staff to rate patients across a series of treatment engagement indicators, measuring the successful completion of treatment programmes (e.g., dialectic behavioral therapy programmes, arts therapies, sexual offending programmes) and linking these to reconviction data after 3- and 6-year follow-ups (this is described in more detail in Section “Data Analysis”.

### Objectives, Aims, and Research Questions

The project's objectives are: (1) to develop a sustainable, secure clinical database that gives us a detailed and valid account of the §63 StGB patient group and (2) to conduct scientific research with the resulting data to answer important clinical and theoretical questions. Within these objectives, specific aims are identified:

Objective One. To develop a sustainable database of the § 63 StGB patient group

1. To develop and maintain a central, secure, nationwide database that stores anonymized clinical, legal and demographic data on patients receiving care under §63 StGB.
2. To hire staff and ensure the administrative capacity necessary to maintain the database.
3. To develop a network of participating forensic hospitals that contribute to this database routinely.
4. To oversee the collection of clinical, legal and demographic data from participating sites every 12 months and reconviction data after 3- and 6-years.

Objective Two. To conduct scientific research on the § 63 StGB patient group data

5. To describe nationwide descriptive statistics and longitudinal trends in clinical, legal, and demographic data for the §63 StGB patient group.
  a. What are the clinical, legal and demographic profiles of §63 StGB patients?
  b. How do the clinical, legal and demographic profiles of §63 StGB patients change over time?
  c. What are the rates of participation in and completion of treatment programs/interventions?
6. To identify demographic, clinical and legal factors associated with leave status and discharge.
  a. Which demographic, clinical and legal variables are associated with leave status and discharge?
  b. Do variables measuring treatment engagement and programme completion predict leave status and discharge?
7. To establish rates of reconviction at 3- and 6-year follow-up intervals and identify to what extent treatment engagement and programme completion predicts reconviction.
  a. What percentage of discharged patients were reconvicted at 3- and 6-year follow-ups?
  b. What was the average time to reconviction?
  c. What types of offenses were committed?
  d. What was the average number of offenses committed at reconviction?
  e. Do variables measuring treatment engagement and programme completion improve our ability to statistically predict reconviction beyond known predictors already identified in the literature (e.g., age, sex, previous criminal convictions)?
8. To identify differences in reconviction and treatment outcomes for women patients and migrant patients.
  a. To what extent do basic clinical, legal and demographic characteristics differ between (i) men and women, and (ii) migrants and non-migrants?
  b. Do rates of participation in and completion of treatment programmes differ between (i) women and men, and (ii) migrants and non-migrants?
  c. Are women convicted of violent crimes more likely to have experienced childhood adversity, such as abuse and neglect, than those convicted of non-violent crimes?
  d. Do rates of reconviction differ between (i) men and women, and (ii) migrants and non-migrants?
  e. Does the average time to reconviction differ between (i) men and women, and (ii) migrants and non-migrants?

## Methods and Analysis

### Anticipated Total Duration of the Project

The total duration of the study will be 8.5 years (102 months). This timeframe is to ensure: (1) the long-term sustainability of the survey, and (2) enough time to conduct a prospective longitudinal study of reconviction rates.

### Pilot Study

A pilot study will take place in the 12 months prior to the first national survey. The purpose of this pilot is to test and revise: the study documents used by participating sites, the §63 StGB survey online interface and data collection and analysis methods. These activities take place in three forensic hospitals. These sites will be able to feedback on their experiences in four ways. First, an email address has been established to respond to any queries or advice about the project. Second, staff will be able to input feedback/suggestions directly into the online survey (a cognitive pre-test). Third, a meeting (in person or over video conference) will be convened between the research team, the clinical directors of each site, and any staff from participating sites who wish to be involved. Fourth, a researcher from Rostock will visit each site to support their data collection in person. Revisions to the project will be made by the research team following this feedback.

### Recruitment

Following the pilot survey, sites will be recruited for the following year's nationwide §63 StGB survey. The reference date is December 31st, data collection will take place through January of each year. Recruitment will then be repeated each project year to recruit new sites. All eligible forensic hospitals in Germany have been or will be asked to participate. Hospitals will be approached in several ways, including through invitation during the national “Bundesdirektorenkonferenz” meetings (meetings of all forensic hospital directors), *via* email and phone contact, through the professional networks of the researchers involved in the project and at relevant conferences. A record of all (non-)participating sites will be maintained to monitor the representativeness of the sample of participating sites.

Project partners at the Clinic for Forensic Psychiatry in Bad Rehburg, the Center for Forensic Psychiatry in Reichenau, University Hospital Bochum, Georg-August-University Göttingen, and University Hospital Ulm will recruit hospitals in their respective states. After the first year, only the sites that did not participate in the previous year will be approached for recruitment purposes; sites that did participate in the previous year will simply be notified of the opening of the new survey window. As of November 2021, 19 out of 64 hospitals treating §63 StGB forensic patients have agreed in writing to participate with nine more expressing interest in principle.

### Sample Size

We estimate a sample size in the pilot year of ~140 patients. This is based on the number of beds and current inpatients at the three pilot sites. The Federal Office of Statistics reported that in 2018 (the most recent year for which we have admission data) there were 5,995 § 63 StGB patients. We estimate that with the inclusion of the four federal states excluded from the Federal Office of Statistics data (representing 13% of the total general population), assuming a similar rate of §63 StGB patents as in the other 12 states (8.2 patients per 100,000), the total number of patients in this group would be *N* = 5,995 + 897 = 6,892. Given (A) the written commitments we have from 19 hospitals to participate, (B) the pilot study sample, and (C) the recruitment rate for the §64 StGB survey (25%; personal communication with survey administrators) we anticipate a sample size in project year 2 approximately *N* = 3,000 patients (~45% of all §63 StGB patients), which we expect to increase through the life of the project. Given admission rates reported by the Federal Office of Statistics in 2018 (19.2%) (4) we estimate an additional 576 admissions in the survey each year. By project year 8 this would total N=6,456 (any patients that are readmitted to §63 StGB treatment in this period are counted again). We estimate that 50% of all participating sites will agree to participate in the reconviction follow-up study. Given that the percentage of the patient group discharged was 23.1% in 2018 (4), we use these considerations to calculate a follow-up sample size of discharged patients at *N* = 546 patients at 3 years; *N* = 745 patients at 6 years. The reconviction sample sizes might be slightly lower if a patient is discharged two or more times in our data collection period.

### Data Collection and Survey Method

Data collection will take place in January every year (project years 2–8) with December 31st as the reference date. This allows sites time alongside other routine activities to comprehensively complete the survey forms. The online survey software will require staff to respond to each question either with accurate data or to indicate that they do not know; this will minimize missing data. The questions included in the §63 StGB survey are all provided as Supplementary Material to this article. Sites will also be able to request pen/paper formats or send data directly *via* password protected spreadsheets. They will be given instructions in a training session and a manual on completing the survey. To minimize the time investment from participating sites, they will be able to use their pre-existing hospital records to aid in the completion of the §63 StGB survey. Although hospital records are subject to the usual limitations of validity and reliability, these will provide good quality data as these are used regularly in patients' treatment and legal planning. Furthermore, we will implement additional checks on data quality (see section Project Governance).

**Figure 1:**
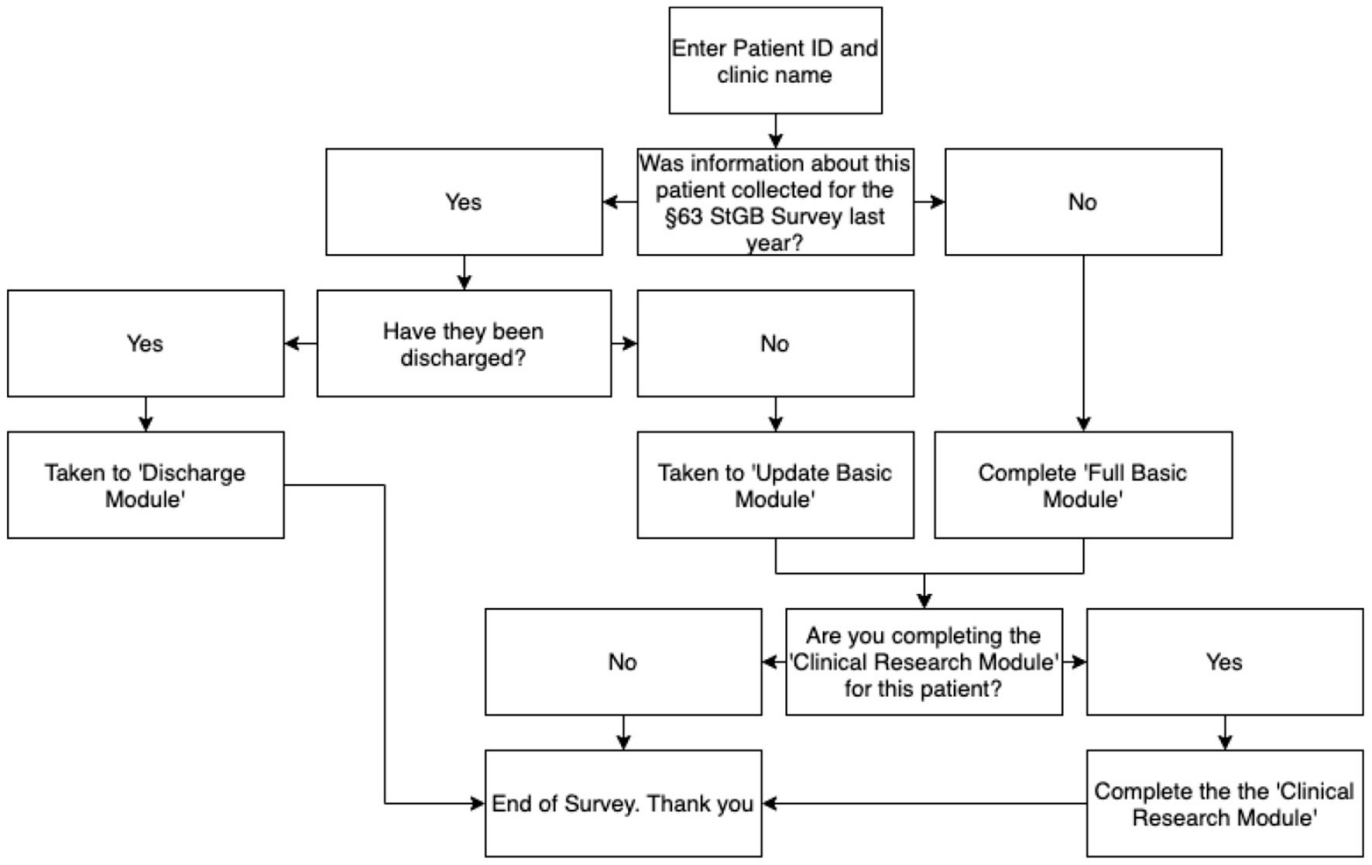
§63 StGB survey algorithm.

Staff based at each participating site will complete surveys for each patient. All patients will be assigned a pseudonymized study ID by staff at each site to facilitate longitudinal data collection and maximize patient anonymity. This survey will be customized to each patient; using four screening questions the online survey will direct staff to the relevant modules automatically. To enable sites to choose their level of involvement and therefore maximize the feasibility and sustainability of the survey and database, sites will choose at which level they have the capacity to participate (See Table 1). The survey logic structure and modules are depicted in Figure 1.

**Table 1:** Participating sites' levels of involvement.

**Level**	**Amount of participation**	**Workload per patient**
1	Complete basic survey for each patient	52 (17) questions
2	Complete basic survey for each patient Complete clinical research module for each patient	52 (17) questions 11 questions
3	Fill in basic survey for each patient Post names for reconviction data	52 (17) questions Spreadsheet for criminal recidivism data
4	Complete basic survey for each patient Complete clinical research module for each patient Post names for reconviction data	52 (17) questions 11 questions Spreadsheet for criminal recidivism data

*Values in brackets show the number of questions to be completed where data have been entered for a patient in the previous years' survey and only the “Update Basic Module” is being completed*.

The survey consists of different modules. The “Full Basic Module” has 52 questions and applies to all newly admitted patients and all patients in the first year of the study. It includes static (historical/background) questions the responses to which will not change over time and dynamic questions where responses will change over time. If data on a patient were included in the previous year's survey, the survey completer will be automatically directed to an “Update Basic Module” with only 17 dynamic questions that need to be updated. This saves time and provides a greater level of respect for patient confidentiality. If data on the patient were included in the previous year's survey but this patient has since been discharged, the survey completer will be automatically directed to a “Discharge Module” with six questions. An optional “Clinical Research” module includes a further eleven detailed questions about patients' dynamic treatment factors.

Sites that participate in the follow-up aspect of the project will submit a document with the information on all patients needed to request reconviction data from the Federal Office of Justice (Bundesamt für Justiz). They will submit this data at the same time but separately from the §63 StGB survey data to ensure patient names and survey data are never handled by the same member of the research team or sent together. The patient data needed to request reconviction data for each individual include: current patient name, name at birth, study code, date of birth, and place of birth. Participating sites will be asked to maintain a record of each patient included in the survey for each year and their study code. This ensures the study is auditable, that data collection processes are transparent, and makes data imputation more efficient for participating sites. Requests for reconviction data will be submitted to the Federal Office of Justice 3- and 6-years after the first national data collection phase (4- and 7-years after the pilot).

### Data Preparation and Missing Data

All data and study documents sent to the Department for Forensic Psychiatry, Rostock University Medical Center will be stored, processed and destroyed appropriately. All data will be checked for completeness or anomalies by a member of the research team. Where anomalies or missing responses have been entered by a participating site (a non-sensical answer, typographical error, etc.) and these cannot be resolved by the research team, the researcher will ask the participating site *via* phone call to resubmit the relevant information by referring to the study ID to which the missing or anomalous data is connected.

### Data Analysis

The statistical software packages IBM SPSS and R will be used to analyse the study data. Significance levels will be two-tailed and set to *p* < 0.05 unless specified. Effect sizes, *p*-values and 95% confidence intervals will be reported. Where multiple comparisons are conducted, the Bonferroni correction (or similar) will be used. Reporting effect sizes, only analyzing data after hypotheses have been generated, and using the Bonferroni correction will help to avoid Type I errors when interpretation findings given the relatively large sample size. Data will be inspected descriptively before analyses are conducted. Normality will be tested with the Shapiro-Wilk statistic. Outliers will be removed from inferential statistics. Statistical methods and analysis are being supervised by statisticians at the Institute for Psychology at the Chemnitz University of Technology.

#### Research Aim 5. to Describe Nationwide Descriptive Statistics and Longitudinal Trends in Clinical, Legal, and Demographic Data for the §63 StGB Patient Group

Basic descriptive statistics will report averages, proportions, percentages and total numbers. Analyses of variance will be conducted to investigate whether longitudinal changes over time are statistically significant. ANOVAs and *t*-tests will be used for data that are normally distributed; Kruskal–Wallis and Mann–Whitney *U*-tests for non-parametric data; Chi-square tests will be used for categorical data in accordance with the procedures described in Navarro (19). Analysis will only proceed where assumptions for these tests are not violated. Appropriate *post-hoc* tests and measures of effect size will be reported. We expect a sample size of *N* = 3,000 in project year 2 and *N* = 6,456 in project year 8, thus, our analyses will be sufficiently powered for these univariate tests.

#### Research Aim 6. to Identify Demographic, Clinical, and Legal Factors Associated With Leave Status and Discharge

To answer questions 6a–b, proportional odds ordinal regression analysis will be conducted with the “R” package “polr” function in the package “MASS” (20, 21). The leave status outcome variable will be coded: (1) no leave, (2) escorted or unescorted access to hospital grounds, (3) escorted leave outside the hospital, (4) unescorted leave outside the hospital, and (5) extra-institutional leave wherein patients stay outside hospital for more than 24 h. A sixth level of the outcome variable, discharge from hospital, will be included in project year 3 as a sufficient number of patients will been discharged by then. Leave status reflects progression through care, i.e., more leave indicates improvement in treatment outcomes. Analysis will only proceed where assumptions are not violated, i.e., proportional odds and multicollinearity.

##### Univariate Analysis

As this analysis is novel in German forensic settings, predictor variables selected based on the international literature and clinical experience will first be entered into univariate ordinal regressions. These include: length of treatment, as leave status changes over time as patients recover; mental health diagnosis, PCL-R score (if available), age at admission, and type of index offense(22, 23)^2^; ratio of the number of adverse events (threatening or aggressive incidents) per month in treatment (or in the previous 12 months); and treatment engagement variables, operationalized as (A) staff-assessed Likert scale questions on participation, responsibility-taking, empathy, insight into disorder and offense/risk factors, and motivation; and (B) number of treatment interventions/programmes successfully completed).

##### Multivariate Analysis

Three hierarchical models will be computed to assess the predictive ability of individual variables and the addition of dynamic treatment variables (Model 3) to a model with static patient characteristics (Model 2). Variables that were significant univariate predictors will be included.

■ Model 1: length of treatment.
■ Model 2: Model 2 will include Model 1 plus mental health diagnosis, age at admission, index offense, PCL-R score (if available), and number of adverse events.
■ Model 3: Model 2 plus treatment engagement and programme completion variables.

Test (likelihood ratio Chi^2^), individual parameter (regression coefficient and S.E.; *t*-value, proportional odds ratios and 95% C.I.), effect size (McFadden *R*^2^), and model fit (AIC, residual deviance) statistics will be reported for all models. As a method of validity-checking, we will analyze the same models using linear regression *via* the “lmer” function in the “lme4” package (24) in “R” and compare findings (the former results will take precedence). Analyses will be conducted each year to increase the power of the models. A multilevel ordinal logistic regression approach will be adopted to account for variation across hospitals when a sufficient number of hospitals for this analysis method are recruited after several years [~50 “level 2” observations are needed (25)].

The literature on required sample size for ordinal logistic regression is not conclusive. Taylor et al. (26) propose that for a model with two predictor variables and an ordinal outcome with five categories to achieve a power of 0.80, a *N* = 224–377 is required. We used G^*^Power (27) to calculate the required sample size for our full analysis if we used linear multiple regression, fixed model, *R*^2^ deviation from zero. Assuming a small effect size *f*^2^ = 0.02, probability = 0.05, power = 0.8 and 13 predictor variables, *N* = 904 observations are needed. Given we expect a sample size of *N* = 3,000 in project year 2 and *N* = 6,456 in project year 8, our analysis will be sufficiently powered. Our data also allow us to investigate (A) to what extent completion of different specific programmes/interventions (e.g., DBT, CBT, psychoanalytical) differentially predict leave and discharge, and (B) which variables predict treatment engagement and programme completion.

#### Research Aim 7. to Establish Rates of Reconviction at 3- and 6-Year Follow-Up Intervals and Identify to What Extent Treatment Engagement and Programme Completion Predicts Reconviction

Basic descriptive statistics will report rates, time until, and types of reconviction (Q7a–d). To answer Q7e, survival analysis, specifically Cox proportional hazards regression with the “coxph” function of the “survival” package in “R” will be conducted (21). The outcome variable is reconviction or not given time-at-risk (time between discharge and offense or study follow-up). Analysis will only proceed where assumptions are not violated, i.e., linearity between continuous predictors and logit of the outcome, independence of errors, and multicollinearity.

##### 3-Year Follow-Up

A Cox proportional hazards analysis will be run with time at risk and reconviction as the outcome. The following variables that have been associated with reoffending in the literature will be added into Model 1: diagnosis, sex, age at discharge, number of previous offenses, and history of substance abuse (28). Test, receiver operating characteristic (ROC) and goodness of fit statistics will be calculated for the model as a whole and ROC values for each predictor variable.

##### 6-Year Follow-Up

The same five predictor variables used in the 3-year follow-up will be entered into Model 1. Given the increased power of the six-year follow-up, variables measuring the ratio of the number of adverse events (threatening or aggressive incidents) per month in treatment and length of treatment will be added to Model 2. The seven variables measuring programme/intervention completion and staff perceptions of treatment engagement will be added in Model 3. Test, ROC and goodness of fit statistics will be observed to assess whether the additional treatment engagement variables improve the statistical properties of Model 1.

Test (likelihood ratio Chi^2^, Wald test, concordance), individual parameter (regression coefficient and S.E.; Wald Z-score; hazard ratios and 95% C.I.), and model fit (AIC) statistics will be reported. ROC curves will be calculated and area under the curve (AUC) values reported for each predictor and model.

To ensure sufficient power for the analysis, we use the rule of thumb described by Peduzzi et al. (23). The authors conclude that the risk of unreliable logistic regression results can occur when the number of events per predictive variable (EPV) is lower than 10. EPV equals the number of events (here: reconvictions)/number of predictor variables. We estimate recruitment rates for the follow-up study as approximately *N* = 546 patients at 3 years; *N* = 745 patients at 6 years (see Section Sample Size). We estimate rates of reconvictions at 10% at the 3-year follow-up and 20% at the 6-year follow-up.^3^ The 3-year follow-up Cox regression Model 1 includes five predictor variables; the EPV = 10.9. The 6-year follow-up Cox regression Model 2 includes 13 predictor variables; the EPV = 11.5. This suggests both analyses will be sufficiently powered and demonstrates the need for a longitudinal study design to recruit a large enough sample to produce novel findings that add to the extant literature. Our data also allow us to investigate to what extent completion of different specific programmes/interventions (e.g., DBT, CBT, psychodynamic therapy) differentially predict reconviction.

#### Research Aim 8. to Identify Differences in Reconviction and Treatment Outcomes for Women Patients and Migrant Patients

To answer Q8a–d, univariate analyses of difference will be conducted. ANOVAs and *t*-tests will be used for data that are normally distributed; Kruskal–Wallis and Mann Whitney *U*-tests for non-parametric data; Chi-square tests will be used for categorical data in accordance with the procedures described in Navarro (19). To answer Q8e, descriptive Kaplan Meier Survival curves will be generated. These will depict the survival (no reconviction) functions for both women and migrant groups compared to men and non-migrants. Significant differences in time to reconviction will be calculated with the Log Rank test (33). We expect a sample size of *N* = 3,000 in project year two and *N* = 6,456 in project year 8, thus, our analyses will be sufficiently powered for these univariate tests.

### Project Governance

Several measures will be taken to ensure the appropriate management and quality assurance of the project. First, an expert advisory panel has been convened to discuss any difficulties that may arise; recommend any necessary improvements to recruitment, data collection, or analysis practices; and monitor the project. This panel comprises international experts in the field of forensic psychiatry with experience in follow-up methods. It will meet with the research team at project workshops in project years one, three and five, and annually *via* video conferencing.

Second, project workshops will be convened in project months 6, 33 and 57 (project years 1, 3, and 5). These will bring together all project partners, the advisory panel, and all forensic hospital directors or their nominees. The purpose of these workshops is to reflect on the project methodology and implementation at participating sites. Third, a project overview file will be compiled. This will log every document pertaining to the project methodology, ethical approvals and amendments thereof, data protection approvals, funding documents, C.V.s and relevant training certificates of the research team, and information on the tasks of each member of the research team. No study data will be stored in this file. All updated versions will be recorded and stored in electronic and paper copies. This will ensure transparency and provide a blueprint if new members join the research team.

Fourth, a project manual and an online training session will be developed. The manual will be a pdf document sent to all participating sites to be passed on to all staff involved in the study. The online training session will be recorded and sent to sites. The manual and workshop will: describe how to use the survey software, how to transfer data to Rostock, how to answer survey questions, give definitions, and provide a list of contact details. This will strengthen the reliability of the survey data by harmonizing staff understanding of how to use the survey and send data.

Finally, we will assess the feasibility of developing a quality assurance mechanism. During the pilot phase and first annual meeting of the expert panel, sites will be asked whether they have the capacity to participate in the following: each year, 5 months after data collection, 5% of participating sites will be randomly selected (each site will be given a number and random numbers will be selected *via* a random number generator). These sites will be asked to complete the static/background questions of the Basic Module for 10% of patients included in the most recent survey again. These data will be sent back to the Department for Forensic Psychiatry, Rostock *via* the online survey or Excel spreadsheet and checked by the research team. Participating sites will be told if, and what percentage of, the data they sent *via* the survey in the quality assurance check did not match the data sent in the most recent survey (i.e., are inconsistent). If this exceeds 5% of the data sent for quality assurance purposes, participating sites will be asked to remind staff of the importance of accuracy when completing the survey. If this exceeds 10% of the data sent for quality assurance purposes, participating sites will also be asked to meet with the research team to discuss the project and be offered support/training in completing the survey. This will be implemented if considered feasible.

## Discussion

This study builds on international movements to routinely measure patient outcomes (34) and develop database infrastructure (9). National efforts to collect such epidemiological data on the §63 StGB patient group in Germany have been undertaken in the past and the present study will complement the current §64 StGB survey study (35, 36). Data collected in this study will be of benefit to patients, carers, healthcare providers, individual clinics, funders, policy-makers and the general public as the findings will tell a story about who is receiving forensic treatment and how this evolves over time with broader legal, social and demographic changes. Collecting data on the number of forensic patients anticipated in this study will allow us to test on an aggregate level whether different groups in care—women patients, patients with a migration background—have different treatment, recovery and criminogenic outcomes. Although this project investigates forensic mental health settings, its findings will be useful in general mental health and prisons. Long-term opportunities connected to this study include linkage with databases in other European countries or more extensive follow-up studies in which discharged patients are interviewed by researchers.

Findings from this project will be situated within the literature generated by other longitudinal studies investigating outcomes in mentally disordered offenders. For example: the retrospective follow-up National Trajectory Project in Canada (37); a 25-year retrospective follow-up of men and women found not guilty by reason of mental illness in Australia (38); and recent register-based retrospective follow-up studies in Sweden, such as a 24-year follow-up of sexual offenders with/without psychotic disorders (39) and a study of post-discharge recidivism for patients admitted to forensic services between 2009 and 2018 (40).

### Limitations

There are several limitations to this study design. First, it is possible that we will not recruit every forensic hospital. This is because clinical settings without dedicated research staff might not have the staffing resources to dedicate to completing the §63 StGB survey. We have sought to ameliorate this problem by making it possible for sites to choose a level of participation they are able to commit to, combine data they provide for their internal databases/surveys with the §63 StGB survey to reduce workload, and by incentivizing sites with a comprehensive report of their results and a comparison with aggregated values from all other sites. Second, data are limited to information already residing within patient notes or conclusions based on evidence in patient notes (e.g., staff assessment of treatment engagement). Clinical records can suffer from a host of problems, including inaccuracies, missing data, incomplete reports, confusing entries, and so forth. This is compounded by the possibility of human error in data input. To address this, we will produce a survey manual and online training session detailing what information is needed for each question to help staff answer survey questions accurately. We will also determine the feasibility of conducting the quality assurance mechanism described in Section ‘Project Governance'. Third, we are using staff assessments of treatment engagement to examine the relationship between this construct and individual outcomes. An analysis that includes patient-reported measures would offer a more detailed examination of this relationship; however, these data are not routinely collected in hospitals and given the nature of the survey methodology and consenting process it is not possible to recruit individual patients to complete measures in this study. Our findings can inform subsequent projects that use patient-reported measures though. Finally, we use reconviction data collected by the Federal Office of Justice in the Federal Central Register (Bundeszentralregister) to evaluate reconviction rates and types. As officially recorded reconviction data, this does not offer a complete overview of all criminal offenses committed by patients included in our follow-up.

## Ethics and Dissemination

### Ethics, Legal Bases, Confidentiality, and Agreement to Participate

This project will be conducted in accordance with ethical and data protection regulations and guidelines. This includes compliance with international law [e.g., General Data Protection Regulation, 2018 (GDPR), and EU Directive 2001/20/EC], national/state law (e.g., the Landeskrankenhausgesetz Mecklenberg-Vorpommern and the Bundeszentralregistergesetz), and guidelines on good clinical practice and clinical research (e.g., the International Conference on Harmonization of Good Clinical Practice, and the Declaration of Helsinki, 2013). We will also adhere to the German Society for Research (DFG) Guidelines on the Handling of Research Data (Adopted by the Senate of the DFG on September 30th, 2015), and the DFG Guidelines for Safeguarding Good Research Practice Code of Conduct (2019). This project has been awarded ethical approval [A 2021-0003] and data protection approvals from the Rostock University Medical Center. We are formalizing our project partnerships through Joint Controller Agreements.

It is not expected that any harm will result for participating sites or patients receiving treatment. Participating forensic psychiatric hospitals will be able to withdraw from the study at any moment. Study sites will provide a signed agreement to participate form. The use of routinely collected patient data without a patient's consent in the context of this study is justified under Art. 6 (1) (e) General Data Protection Regulation (GDPR) in conjunction with Art. 9 (2) (j) GDPR, with respect to the safeguards described in Art. 89 (1) GDPR. This is further based on §37 Landeskrankenhausgesetz (LKHG M-V). The collection of criminal records will be conducted in accordance with Art. 10 GDPR. The legal basis for requesting the reconviction data from the Federal Central Register is §42a Bundeszentralregistergesetz (BZRG).

The method of obtaining agreement to participate from participating sites instead of individual patients *via* the use of survey methods to collect demographic, clinical and legal data on forensic patients from existing clinical records was approved from the Ministry of Education, Science, and Culture Mecklenburg-Western Pomerania. This approach is usual for such research designs as attempting to collect individual consent from all individual patients would substantially limit the scope of the study and skew the sample, thus affecting the validity of the findings. Patients' interests, legal status or treatment will not be impaired. This method allows a larger and more representative sample to be collected, which has important implications as there exists significant public interest in the treatment and outcomes of this patient group.

The §63 Survey data collected in the study are pseudonymized, routinely collected by hospitals, and do not require any interactions with or contributions from patients. The data collected to request the reconviction data from the Federal Office of Justice will not be pseudonymized when they are sent to Rostock but will be pseudonymized after the Federal Office of Justice returns the reconviction data to Rostock in accordance with Federal Office of Justice regulations. No clinical, legal or criminological data are sent to the Federal Office of Justice when requesting the reconviction data. The data collected to request the reconviction data from the Federal Office of Justice are also routinely collected and every effort is made to ensure the data are pseudonymized as soon as possible. No patient names will be included in any documentation alongside §63 Survey data or reconviction data, only study codes will connect these. Every effort has been undertaken to ensure GDPR-compliance.

All data will be treated confidentially by the research team. The results of the survey and analyses will be published in publicly accessible formats (scientific publications, reports, presentations). These publicly published results will be at aggregated group-level and it will not be possible to identify any individual patient or hospital (participating site). No patient study codes will be included in any publications or presentations. We have adopted data protection measures so that we adhere to best practices in line with the “motivated intruder” test (41). Given that no member of staff will have access to personally identifiable data (e.g., patient name, date of birth) and §63 StGB survey or reconviction data, that participating sites are asked to not send data describing highly identifiable/publicly known patients, and the use of pseudonymized patient codes, it will not be possible for staff at Rostock or any agent who illegally acquires the data (motivated intruder) to identify a specific patient based on the data they have access to or that have previously been made public.

### Dissemination and Data Accessibility

Our results will be published in open-access peer-reviewed journals and in the form of publicly available annual reports. Each participating hospital will be sent a report of the results from their site alongside national averages. The results will also be shared at academic conferences and on social media, including Twitter and the blog of the Department for Forensic Psychiatry, Rostock University Medical Center. All project partners will be sent study findings.

We will establish a committee of experts to review requests from individual researchers based at other institutes to access the data collected in this study. These experts will include members of the research team at the Department for Forensic Psychiatry, Rostock University Medical Center, project partners, and other suitable individuals, such as clinical directors or head psychologists at forensic hospitals. Applications to access the data (excluding criminal record data) will be reviewed by at least two members of the committee, chosen by the head of the committee (chief investigator, BV). To ensure ethical and data protection compliance, only clearly specified, anonymized and limited data will be sent to external researchers who can articulate the following:

■ Research questions/aims of the study.
■ Hypotheses (if relevant).
■ Which specific variables (e.g., age, diagnosis, index offense) are required for the study.
■ Plans for data protection, storage and management (including plans to delete the data).
■ Whether local ethical approval has/will be obtained.
■ Whether local data protection approval has/will be obtained.

## The CONNECT Study Group

CONNECT, COllaboration to establish a NatioNal databasE on the Criminological and Treatment outcomes of forensic psychiatric patients in Germany. The CONNECT Study Group includes: Jack Tomlin^1^, Peggy Walde^2^, Birgit Völlm^2^, Dörte Berthold^3^, Christian Riedemann^3^, Thomas Ross^4^, Jan Bulla^4^, Boris Schiffer^5^, Manuela Dudeck^6^, Isabell Winkler^7^, Markus Burkhardt^7^, and Jürgen L. Müller^8^

^1^School of Law and Criminology, University of Greenwich, London, United Kingdom

^2^Department for Forensic Psychiatry, Rostock University Medical Center, Rostock, Germany

^3^Clinic for Forensic Psychiatry, Bad Rehburg, Germany

^4^Department of Forensic Psychiatry and Psychotherapy, Centre of Psychiatry, Reichenau, Germany

^5^Clinic for Forensic Psychiatry in Herne, Department of Forensic Psychiatry and Psychotherapy, LWL-University Hospital Bochum, Ruhr-University Bochum, Bochum, Germany

^6^University Hospital Ulm and Clinic for Forensic Psychiatry in Günzburg, Günzburg, Germany

^7^Institute for Psychology, Chemnitz University of Technology, Chemnitz, Germany

^8^Clinic for Psychiatry and Psychotherapy—Forensic Psychiatry, Human Medical Center Göttingen, Georg-August-University Göttingen, Göttingen, Germany

## Author Contributions

JT, PW, and BV contributed equally to the conception and design of the study and have contributed to the authorship of the manuscript over various iterations. The CONNECT Study Group have contributed to the development of the study design or research methods. All authors have approved the submitted version.

## Funding

This project has received financial support from the Rostock University Medical Center FORUN-Program for Junior Scientists [Antrags-Nr.: 889038].

## Conflict of Interest

The authors declare that the research was conducted in the absence of any commercial or financial relationships that could be construed as a potential conflict of interest.

## Publisher's Note

All claims expressed in this article are solely those of the authors and do not necessarily represent those of their affiliated organizations, or those of the publisher, the editors and the reviewers. Any product that may be evaluated in this article, or claim that may be made by its manufacturer, is not guaranteed or endorsed by the publisher.

## Footnotes

^1^The official translation of §63 StGB reads as follows: If a person has committed an unlawful act in a state of criminal irresponsibility (section 20) or in a state of diminished responsibility (section 21), the court orders that person's placement in a psychiatric hospital if the overall evaluation of the offender and of the offence reveals that, due to the offender's condition, he or she represents a danger to the general public on account of it being expected that he or she will in future commit serious unlawful acts which will result in the victims of the offence suffering or being exposed to the considerable danger of severe emotional trauma or physical injury or which will cause serious economic damage. If the unlawful act which has been committed is not an offence as referred to in sentence 1, the court only makes such an order if special circumstances justify the expectation that, due to the offender's condition, the offender will in future commit such serious offences.

^2^Clercx M, Verhees M, Keulen de Vos ME. *Forensic Offenders and the Steps of Re-Entry Back Into Society: The Relationship Between Static Patient Characteristics and Leave During Mandated Stay in a Forensic Hospital* (Under review).

^3^We used past follow-up studies of § 63 StGB patients to estimate reoffending rates (events) at 3- and 6-year intervals. These rates are: 5% at 12 months (29); 7% at 12 months (30); 6% at Ø12.6 months22; 16.5% at 3.9 years and 25.2% at 7.5 years (31); 31.2% at 5 years (32).
