# Supplementary material for: Protocol for the CONNECT Study: A National Database and Prospective Follow-Up Study of Forensic Mental Health Patients in Germany
Source: Front Psychiatry. 2022 Apr 25;13:827272. doi: 10.3389/fpsyt.2022.827272 (PMC9081526; doi:10.3389/fpsyt.2022.827272)
Supplement: Supplementary file 1 [file Data_Sheet_1.PDF]

FORENSIK

ROSTOCK

Rostock § 63 StGB Survey
The CONNECT Study

Jack Tomlin, Peggy Walde, Birgit Völlm, and the CONNECT Study Group

The CONNECT Study Group includes:

Jack Tomlin[1], Peggy Walde[2], Birgit Völlm[2], Dörte Berthold[3], Christian Riedemann[3], Thomas Ross[4], Jan Bulla[4], Boris Schiffer[5], Manuela Dudeck[6], Isabell Winkler[7], Markus Burkhardt[7], and Jürgen L. Müller[8]

[1]School of Law and Criminology, University of Greenwich, London, UK
[2]Department for Forensic Psychiatry, Rostock University Medical Center, Germany
[3]Clinic for Forensic Psychiatry, Bad Rehburg, Germany
[4]Department of Forensic Psychiatry and Psychotherapy, Centre of Psychiatry, Reichenau, Germany
[5]Clinic for Forensic Psychiatry in Herne, and Department of Forensic Psychiatry and Psychotherapy, LWL-University Hospital Bochum, Ruhr-University Bochum
[6]University Hospital Ulm and Clinic for Forensic Psychiatry in Günzburg
[7]Institute for Psychology, Chemnitz University of Technology, Germany
[8]Clinic for Psychiatry and Psychotherapy - Forensic Psychiatry, Human Medical Center Göttingen, Georg-August-University Göttingen, Göttingen, Germany

Inhalt

Screening-Fragen (4 Fragen)......3
Basismodul (52 Fragen)......4
Basismodul aktualisieren (17 Fragen)......20
Klinisches Forschungsmodul (11 Fragen)......30
Entlassungsmodul (8 Fragen)......41

Screening-Fragen (4 Fragen)

1. **Klinik:** ____

2. **Patienten-ID:** ____

*(Codierung der PI-Nr: 3. Buchstabe des Vornamens, 3. Buchstabe des Nachnamens, Tag des Geburtstags, die letzten 2 Zahlen des Geburtsjahres - z. B.: Peter Müller, geb. 18.05.1973 = TL1873)

3. **Wurden Informationen zu dieser*m Patient*in bereits im letzten Jahr bei der §63 StGB Stichtagserhebung erhoben?**

   a. Ja (-> Frage 4)
   b. Nein (-> Basismodul)

4. **Wurde diese*r Patient*in in den letzten 12 Monaten entlassen?**

   a. Ja (-> Entlassungsmodul)
   b. Nein (-> Basismodul aktualisieren)

Basismodul (52 Fragen)

Soziodemografische Angaben

1. Geschlecht

☐ Weiblich
☐ Männlich
☐ Divers
☐ Unbekannt

2. Alter am Stichtag [z.B. 31.12.2022]

____
☐ Unbekannt

3. Geburtsland

☐ Deutschland
☐ Sonstige:

____
☐ Unbekannt

4. Aktueller Beziehungsstatus

☐ Ledig
☐ Verheiratet / in eingetragener Partnerschaft
☐ In sonstiger fester Partnerschaft
☐ Verwitwet
☐ Geschieden
☐ Unbekannt

5. Hat der/die Patient*in die Personensorge oder Teile dieser für ein oder mehrere Kinder?

☐ Ja
☐ Nein
☐ Unbekannt

6. Migrationshintergrund

Eine Person hat dann einen Migrationshintergrund, wenn sie selbst oder mindestens ein Elternteil nicht mit deutscher Staatsangehörigkeit geboren ist.

☐ Kein Migrationshintergrund
☐ Ja - in Deutschland geboren
☐ Ja - aus dem Ausland zugewandert Verheiratet
☐ Unbekannt

7. Staatsangehörigkeit

☐ Deutsch
☐ Sonstige:
____
☐ Unbekannt

8. Ist der/die Patient*in Spätaussiedler?

Spätaussiedler sind nach der gesetzlichen Definition des Bundesvertriebenengesetzes deutsche Volkszugehörige, die die Republiken der ehemaligen Sowjetunion nach dem 31. Dezember 1992 im Wege des Aufnahmeverfahrens verlassen und sich innerhalb von sechs Monaten in Deutschland niedergelassen haben.

☐ Ja
☐ Nein
☐ Unbekannt

9. Schulbildung am Stichtag [z.B. 31.12.2022]

☐ Förderschule
☐ Hauptschule
☐ Realschule/POS
☐ Abitur/Fachabitur
☐ Keine
☐ Unbekannt

10. Berufsausbildung am Stichtag [z.B. 31.12.2022]

☐ Ungelernt/abgebrochene Berufsausbildung
☐ Abgeschlossene Berufsausbildung
☐ Abgeschlossenes Studium (FH/Universität)
☐ Keine
☐ Unbekannt

11. Arbeitstätigkeit zum Zeitpunkt des Anlassdeliktes

☐ Sozialversicherungspflichtige Tätigkeit in Voll- oder Teilzeit
☐ Gelegenheitsarbeit /Minijob (gelegentliche / geringfügige, sozialversicherungspflichtige Beschäftigung)
☐ Ausbildung in Voll- oder Teilzeit
☐ Studium in Voll- oder Teilzeit
☐ Praktikum
☐ Selbstständig
☐ Berentet
☐ Sonstiges
☐ Unbekannt

12. Lebenssituation bei der Anlassstraftat

☐ Allein lebend
☐ Bei den Eltern/Sorgeberechtigten lebend

☐ Mit Partner*in zusammen lebend
☐ Wohngemeinschaft
☐ Betreutes Wohnen
☐ Übergangswohnheim/Wohnheim
☐ Wohnungslos
☐ Psychiatrisches Krankenhaus
☐ Justizvollzugsanstalt
☐ Sonstiges
☐ Unbekannt

Angaben zur Anamnese

13. Schädlicher Gebrauch / Abhängigkeit von Alkohol bei mindestens einer sorgeberechtigten Person in der Kindheit

Mehrfachnennung möglich
☐ Biologischer Elternteil (diagnostiziert bzw. wahrscheinlich)
☐ Nicht-biologische sorgeberechtigte Person / Elternteil (diagnostiziert bzw. wahrscheinlich)
☐ Nein
☐ Unbekannt

14. Schädlicher Gebrauch / Abhängigkeit von anderen Substanzen (inkl. Medikamente) bei mindestens einer sorgeberechtigten Person in der Kindheit

Mehrfachnennung möglich
☐ Biologischer Elternteil (diagnostiziert bzw. wahrscheinlich)
☐ Nicht-biologische sorgeberechtigte Person / Elternteil (diagnostiziert bzw. wahrscheinlich)
☐ Nein
☐ Unbekannt

15. Hinweise auf eine andere schwere psychische Erkrankung bei mindestens einer sorgeberechtigten Person in der Kindheit

Mehrfachnennung möglich
☐ Biologischer Elternteil (diagnostiziert bzw. wahrscheinlich)
☐ Nicht-biologische sorgeberechtigte Person / Elternteil (diagnostiziert bzw. wahrscheinlich)
☐ Nein
☐ Unbekannt

16. Hinweise auf Delinquenz bei mindestens einer sorgeberechtigten Person in der Kindheit

Mehrfachnennung möglich
☐ Biologischer Elternteil (verurteilt oder wahrscheinlich)
☐ Nicht-biologische sorgeberechtigte Person / Elternteil (verurteilt oder wahrscheinlich)
☐ Nein

☐ Unbekannt

17. Emotionaler Missbrauch oder Vernachlässigung in der Kindheit

☐ Ja
☐ Nein
☐ Unbekannt

18. Körperlicher Missbrauch in der Kindheit

☐ Ja
☐ Nein
☐ Unbekannt

19. Sexueller Missbrauch in der Kindheit

☐ Ja
☐ Nein
☐ Unbekannt

20. Jemals Suizidversuche (vor dem aktuellen Aufenthalt)

☐ Ja
☐ Nein
☐ Unbekannt

21. Jemals selbstverletzendes Verhalten (vor dem aktuellen Aufenthalt)

☐ Ja
☐ Nein
☐ Unbekannt

22. Vorige stationäre allgemeinpsychiatrische Behandlung

☐ Nein
☐ 1-mal
☐ 2-5-mal
☐ 6-9-mal
☐ 10-mal oder mehr
☐ Ja, aber Anzahl unbekannt
☐ Unbekannt

23. Vorige stationäre suchtmedizinische Behandlung

☐ Nein
☐ 1-mal
☐ 2-5-mal
☐ 6-9-mal
☐ 10-mal oder mehr
☐ Ja, aber Anzahl unbekannt

☐ Unbekannt

24. Aufnahmedatum in aktuelle Klinik
(in Monat / Jahr, z. B.: 03/2016)

____
☐Unbekannt

25. Aufnahmedatum für die aktuelle Maßregelvollzugsbehandlung

(Einschließlich Aufenthalt in anderen Maßregelvollzugseinrichtungen, falls Patient*in direkt aus einem anderen Maßregelvollzug übernommen wurde)
(in Monat / Jahr, z. B.: 03/2016)

____
☐ Gleich wie Aufnahmedatum aktuelle Klinik
☐ Unbekannt

26. Aufnahme aus:

☐ Polizeigewahrsam
☐ Justizvollzugsanstalt
☐ Jugendhaftanstalt
☐ Andere Maßregelvollzugseinrichtung
☐ Allgemeinpsychiatrische Klinik
☐ Suchthilfeeinrichtung
☐ Allgemeines Krankenhaus
☐ Sonstige Einrichtung/Institution

____
☐ Eigene Häuslichkeit
☐ Unbekannt

Strafrechtliche Dokumentation

27. Beurteilung der Schuldfähigkeit im Urteil des Anlassdeliktes

☐ § 20 StGB
☐ § 21 StGB
☐ Unbekannt

28. Zusätzlich angeordnete Haftstrafe

☐ Ja
☐ Nein
☐ Unbekannt

29. Falls zutreffend, Dauer in Monaten

(Bitte nur ganze Zahlen angeben, z.B. „22“)

____
☐ Lebenslang
☐ Unbekannt

30. Anzahl der Einträge im Bundeszentralregister (BZR) bis zum Erhebungszeitpunkt einschließlich der Anlassstraftat

____
☐ Unbekannt

31. Anzahl verbüßter Haftstrafen

____
☐ Keine
☐ Unbekannt

32. Alter bei der ersten verurteilten Straftat

____
☐ Unbekannt

33. Alter bei der ersten Haftstrafe

____
☐ Unbekannt

34. Dauer der längsten vergangenen Haftstrafe in Monaten

Wenn Daten nur für Jahre verfügbar sind, multiplizieren Sie Jahre x 12 (Monaten)

Monate: ___________________________
☐ Unzutreffend
☐ Unbekannt

35. Vorige Unterbringung in einer Maßregelvollzugseinrichtung

(außer aktuelles Verfahren)
☐ Nein
☐ 1-mal
☐ 2-mal
☐ 3-mal oder mehr
☐ Unbekannt

36. Gesamtlänge Unterbringung Maßregelvollzug vor der jetzigen Unterbringung

Wenn Daten nur für Jahre verfügbar sind, multiplizieren Sie Jahre x 12 (Monaten)
Monate: ___________________________

☐ Unzutreffend
☐ Unbekannt

37. Anlassstraftat

(Mehrfachnennung möglich)
☐ Tötungsdelikt
☐ Versuchtes Tötungsdelikt
☐ Sonstiges Körperverletzungsdelikt
☐ Sexualdelikt gegen Erwachsene (inkl. mit Todesfolge)
☐ Sexualdelikt gegen Minderjährige (inkl. mit Todesfolge)
☐ Sonstiges Gewaltdelikt (z. B. Raub, Erpressung, Nötigung, Bedrohung)
☐ Eigentumsdelikt (z. B. Diebstahl, Betrug)
☐ Brandstiftung
☐ Verkehrsdelikt
☐ BtMG-Delikt
☐ Weisungsverstoß
☐ Sonstiges __________
☐ Unbekannt

38. Vergangene Straftaten (vor der Anlassstraftat)

(Mehrfachnennung möglich)
☐ Tötungsdelikt
☐ Versuchtes Tötungsdelikt
☐ Sonstiges Körperverletzungsdelikt
☐ Sexualdelikt gegen Erwachsene (inkl. mit Todesfolge)
☐ Sexualdelikt gegen Minderjährige (inkl. mit Todesfolge)
☐ Sonstiges Gewaltdelikt (z. B. Raub, Erpressung, Nötigung, Bedrohung)
☐ Eigentumsdelikt (z. B. Diebstahl, Betrug)
☐ Brandstiftung
☐ Verkehrsdelikt
☐ BtMG-Delikt
☐ Weisungsverstoß
☐ Sonstiges __________
☐ Unbekannt

39. Hauptopfer oder Geschädigter durch Anlassstraftat

(Mehrfachnennung möglich)
☐ Mutter (inkl. Pflege-)
☐ Vater (inkl. Pflege-)
☐ Geschwister (einschl. Stiefgeschwister)
☐ Intimpartner*in
☐ Eigene Kinder, erwachsen (einschliesslich Pflege-)
☐ Eigene Kinder, minderjährig (einschliesslich Pflege-)
☐ Andere Familienangehörige

☐ Keine Beziehung zum Täter*in
☐ Bekannte*r/Freund*in
☐ Firma, Organisation, Staat
☐ Sonstiges
☐ Unbekannt

Diagnosen und Behandlung

40. Aktuelle psychiatrische Diagnosen

**Diagnose**	**Ja**	**ICD-10-Kode**
F00-F09 Organische, einschließlich symptomatischer psychischer Störungen	☐	☐ F00. Demenz bei Alzheimer-Krankheit<br>☐ F01. Vaskuläre Demenz<br>☐ F02. Demenz bei anderenorts klassifizierten Krankheiten<br>☐ F03. Nicht näher bezeichnete Demenz<br>☐ F04. Organisches amnestisches Syndrom, nicht durch Alkohol oder andere psychotrope Substanzen bedingt<br>☐ F05. Delir, nicht durch Alkohol oder andere psychotrope Substanzen bedingt<br>☐ F06. Andere psychische Störungen aufgrund einer Schädigung oder Funktionsstörung des Gehirns oder einer körperlichen Krankheit<br>☐ F07. Persönlichkeits- und Verhaltensstörung aufgrund einer Krankheit, Schädigung oder Funktionsstörung des Gehirns<br>☐ F09. Nicht näher bezeichnete organische oder symptomatische psychische Störung
F10-F19 Psychische und Verhaltensstörungen durch psychotrope Substanzen	☐	☐ F10. Psychische und Verhaltensstörungen durch Alkohol<br>☐ F11. Psychische und Verhaltensstörungen durch Opioide<br>☐ F12. Psychische und Verhaltensstörungen durch Cannabinoide<br>☐ F13. Psychische und Verhaltensstörungen durch Sedativa oder Hypnotika<br>☐ F14. Psychische und Verhaltensstörungen durch Kokain<br>☐ F15. Psychische und Verhaltensstörungen durch andere Stimulanzien, einschließlich Koffein<br>☐ F16. Psychische und Verhaltensstörungen durch Halluzinogene<br>☐ F17. Psychische und Verhaltensstörungen durch Tabak<br>☐ F18. Psychische und Verhaltensstörungen durch

		flüchtige Lösungsmittel<br>☐ F19. Psychische und Verhaltensstörungen durch multiplen Substanzgebrauch und Konsum anderer psychotroper Substanzen
F20-F29 Schizophrenie, schizotype und wahnhafte Störungen	☐	☐ F20. Schizophrenie<br>☐ F21. Schizotype Störung<br>☐ F22. Anhaltende wahnhafte Störungen<br>☐ F23. Akute vorübergehende psychotische Störungen<br>☐ F24. Induzierte wahnhafte Störung<br>☐ F25. Schizoaffektive Störungen<br>☐ F28. Sonstige nichtorganische psychotische Störungen<br>☐ F29. Nicht näher bezeichnete nichtorganische Psychose
F30-F39 Affektive Störungen	☐	☐ F30. Manische Episode<br>☐ F31. Bipolare affektive Störung<br>☐ F32. Depressive Episode<br>☐ F33. Rezidivierende depressive Störung<br>☐ F34. Anhaltende affektive Störungen<br>☐ F38. Andere affektive Störungen<br>☐ F39. Nicht näher bezeichnete affektive Störung
F40-F48 Neurotische, Belastungs- und somatoforme Störungen	☐	☐ F40. Phobische Störungen<br>☐ F41. Andere Angststörungen<br>☐ F42. Zwangsstörung<br>☐ F43. Reaktionen auf schwere Belastungen und Anpassungsstörungen<br>☐ F44. Dissoziative Störungen [Konversionsstörungen]<br>☐ F45. Somatoforme Störungen<br>☐ F48. Andere neurotische Störungen
F50-F59 Verhaltensauffälligkeiten mit körperlichen Störungen und Faktoren	☐	☐ F50. Essstörungen<br>☐ F51. Nichtorganische Schlafstörungen<br>☐ F52. Sexuelle Funktionsstörungen, nicht verursacht durch eine organische Störung oder Krankheit<br>☐ F53. Psychische oder Verhaltensstörungen im Wochenbett, anderenorts nicht klassifiziert<br>☐ F54. Psychologische Faktoren oder Verhaltensfaktoren bei anderenorts klassifizierten Krankheiten<br>☐ F55. Schädlicher Gebrauch von nichtabhängigkeitserzeugenden Substanzen

		☐ F59. Nicht näher bezeichnete Verhaltensauffälligkeiten bei körperlichen Störungen und Faktoren
F60-F69 Persönlichkeits- und Verhaltensstörungen	☐	☐ F60. Spezifische Persönlichkeitsstörung<br>☐ F60.0. Paranoide Persönlichkeitsstörung<br>☐ F60.1. Schizoide Persönlichkeitsstörung<br>☐ F60.2. Dissoziale Persönlichkeitsstörung<br>☐ F60.3. Emotional instabile Persönlichkeitsstörung<br>☐ F60.4. Histrionische Persönlichkeitsstörung<br>☐ F60.5. Anankastische [zwanghafte] Persönlichkeitsstörung<br>☐ F60.6. Ängstliche (vermeidende) Persönlichkeitsstörung<br>☐ F60.7. Abhängige (asthenische) Persönlichkeitsstörung<br>☐ F60.8. Sonstige spezifische Persönlichkeitsstörungen<br>☐ F60.9. Persönlichkeitsstörung, nicht näher bezeichnet<br>☐ F61. Kombinierte und andere Persönlichkeitsstörungen<br>☐ F62. Andauernde Persönlichkeitsänderungen, nicht Folge einer Schädigung oder Krankheit des Gehirns<br>☐ F63. Abnorme Gewohnheiten und Störungen der Impulskontrolle<br>☐ F64. Störungen der Geschlechtsidentität<br>☐ F65. Störungen der Sexualpräferenz<br>☐ F66. Psychische und Verhaltensstörungen in Verbindung mit der sexuellen Entwicklung und Orientierung<br>☐ F68. Andere Persönlichkeits- und Verhaltensstörungen<br>☐ F69. Nicht näher bezeichnete Persönlichkeits- und Verhaltensstörung
F70-F79 Intelligenzminderung	☐	☐ F70. Leichte Intelligenzminderung<br>☐ F71. Mittelgradige Intelligenzminderung<br>☐ F72. Schwere Intelligenzminderung<br>☐ F73. Schwerste Intelligenzminderung<br>☐ F74. Dissoziierte Intelligenz<br>☐ F78. Andere Intelligenzminderung<br>☐ F79. Nicht näher bezeichnete Intelligenzminderung
F80-F89 Entwicklungsstörungen	☐	☐ F80. Umschriebene Entwicklungsstörungen des Sprechens und der Sprache<br>☐ F81. Umschriebene Entwicklungsstörungen schulischer Fertigkeiten<br>☐ F82. Umschriebene Entwicklungsstörung der

		motorischen Funktionen<br>☐ F83. Kombinierte umschriebene Entwicklungsstörungen<br>☐ F84. Tief greifende Entwicklungsstörungen<br>☐ F88. Andere Entwicklungsstörungen<br>☐ F89. Nicht näher bezeichnete Entwicklungsstörung
F90-F98 Verhaltens- und emotionale Störungen mit Beginn in der Kindheit und Jugend	☐	☐ F90. Hyperkinetische Störungen<br>☐ F91. Störungen des Sozialverhaltens<br>☐ F92. Kombinierte Störung des Sozialverhaltens und der Emotionen<br>☐ F93. Emotionale Störungen des Kindesalters<br>☐ F94. Störungen sozialer Funktionen mit Beginn in der Kindheit und Jugend<br>☐ F95. Ticstörungen<br>☐ F98. Andere Verhaltens- und emotionale Störungen mit Beginn in der Kindheit und Jugend
F99-F99 Nicht näher bezeichnete psychische Störungen	☐	☐ F99. Psychische Störung ohne nähere Angabe

41. Intelligenzquotient (IQ)

Ergebnis: _______________
Fragebogen zur Bestimmung (falls bekannt): _______________
☐ Unbekannt

42. Falls psychische und Verhaltensstörungen durch psychotrope Substanzen (F10-F19), welches ist/sind die Hauptkonsummittel?

(Mehrfachnennung möglich, bis zu drei Nennungen)
☐ Alkohol
☐ Opioide
☐ Cannabinoide
☐ Sedativa oder Hypnotika
☐ Kokain
☐ Andere Stimulanzien
☐ Halluzinogene
☐ Flüchtige Lösungsmittel
☐ Medikamente
☐ Amphetamine
☐ NPS (Neue psychoaktive Substanzen)
☐ Sonstiges ____________________________
☐ Unbekannt

43. Aktuelle somatische Diagnosen

(Mehrfachnennung möglich)
☐ Bluthochdruck
☐ Diabetes
☐ Sonstige Herz-Kreislauf-Erkrankungen
☐ Adipositas (BMI> 30)
☐ Krebs
☐ Epilepsie
☐ Erkrankung der Atemwege
bitte angeben ________________________
☐ Erkrankung des Magen-Darm-Systems
bitte angeben ________________________
☐ Erkrankung des Bewegungsapparates
bitte angeben ________________________
☐ Infektionserkrankungen (z.B. HIV, Hepatitis)
bitte angeben ________________________
☐ Andere relevante Erkrankungen
bitte angeben ________________________
☐ Keine
☐ Unbekannt

44. Raucher*in?

☐ Ja
☐ Nein
☐ Unbekannt

45. Art der derzeitigen Station

☐ Aufnahmestation
☐ Hochgesicherte Station
☐ Reguläre Behandlungsstation
☐ Long-Stay-Station
☐ Station zur Entlassungsvorbereitung
☐ Außenwohnstätte
☐ Langzeitbeurlaubung
☐ Sonstige ________________________
☐ Unbekannt

46. Vorfälle in den letzten 12 Monaten der Maßregelvollzugsbehandlung

Mehrfachnennung möglich. Fragen zu Fluchten kommen später in der Umfrage.

a. Vorfälle in der Klinik

☐ Nein, keine

☐ verbale Aggression gegenüber Mitarbeiter*innen — Wenn ‚Ja‘, wie häufig? __________

☐ körperliche Übergriffe gegenüber Mitarbeiter*innen	Wenn ‚Ja', wie häufig?	__________
☐ Sexuelle Übergriffe gegenüber Mitarbeiter*innen	Wenn ‚Ja', wie häufig?	__________
☐ verbale Aggression gegenüber Patienten*innen	Wenn ‚Ja', wie häufig?	__________
☐ körperliche Übergriffe gegenüber Patienten*innen	Wenn ‚Ja', wie häufig?	__________
☐ Sexuelle Übergriffe gegenüber Patienten*innen	Wenn ‚Ja', wie häufig?	__________
☐ Sachbeschädigung	Wenn ‚Ja', wie häufig?	__________
☐ Brandstiftung	Wenn ‚Ja', wie häufig?	__________
☐ Substanzkonsum	Wenn ‚Ja', wie häufig?	__________
☐ Scheitern Langzeitbeurlaubung/ Probewohnen	Wenn ‚Ja', wie häufig?	__________
☐ Sonstiges ______________________	Wie häufig?	__________

☐ Unbekannt

b. Vorfälle während Ausgang (begleiteter oder unbegleiteter)

☐ Keine

☐ verbale Aggression gegenüber Mitarbeiter*innen	Wenn ‚Ja', wie häufig?	__________
☐ körperliche Übergriffe gegenüber Mitarbeiter*innen	Wenn ‚Ja', wie häufig?	__________
☐ Sexuelle Übergriffe gegenüber Mitarbeiter*innen	Wenn ‚Ja', wie häufig?	__________
☐ verbale Aggression gegenüber Patienten*innen	Wenn ‚Ja', wie häufig?	__________
☐ körperliche Übergriffe gegenüber Patienten*innen	Wenn ‚Ja', wie häufig?	__________

☐ Sexuelle Übergriffe gegenüber Patienten*innen | Wenn ‚Ja', wie häufig? __________

☐ Sachbeschädigung | Wenn ‚Ja', wie häufig? __________

☐ Brandstiftung | Wenn ‚Ja', wie häufig? __________

☐ Substanzkonsum | Wenn ‚Ja', wie häufig? __________

☐ Sonstiges ______________________ | Wie häufig? __________

☐ Unbekannt

c. Vorfälle während Langzeitbeurlaubung/Probewohnen

☐ Keine

☐ verbale Aggression gegenüber Mitarbeiter*innen | Wenn ‚Ja', wie häufig? __________

☐ körperliche Übergriffe gegenüber Mitarbeiter*innen | Wenn ‚Ja', wie häufig? __________

☐ Sexuelle Übergriffe gegenüber Mitarbeiter*innen | Wenn ‚Ja', wie häufig? __________

☐ verbale Aggression gegenüber Patienten*innen | Wenn ‚Ja', wie häufig? __________

☐ körperliche Übergriffe gegenüber Patienten*innen | Wenn ‚Ja', wie häufig? __________

☐ Sexuelle Übergriffe gegenüber Patienten*innen | Wenn ‚Ja', wie häufig? __________

☐ Sachbeschädigung | Wenn ‚Ja', wie häufig? __________

☐ Brandstiftung | Wenn ‚Ja', wie häufig? __________

☐ Substanzkonsum | Wenn ‚Ja', wie häufig? __________

☐ Sonstiges ______________________ | Wie häufig? __________

☐ Unbekannt

i. Falls ‚ja', führte dies zu einer Wiederaufnahme in die Klinik?

☐ Ja
☐ Nein
☐ Unbekannt

47. Strafrechtliche Verurteilungen für im Maßregelvollzug begangene Straftaten in den letzten 12 Monaten

(Mehrfachnennung möglich; Klinik, Ausgang und Langzeitbeurlaubung/Probewohnen)

☐ Tötungsdelikt	Wenn ‚Ja', wie häufig?	___________
☐ Versuchtes Tötungsdelikt	Wenn ‚Ja', wie häufig?	___________
☐ Sonstiges Körperverletzungsdelikt	Wenn ‚Ja', wie häufig?	___________
☐ Sexualdelikt gegen Erwachsene	Wenn ‚Ja', wie häufig?	___________
☐ Sexualdelikt gegen Kinder	Wenn ‚Ja', wie häufig?	___________
☐ Sonstiges Gewaltdelikt (z.B. Raub, Erpressung, Nötigung, Bedrohung)	Wenn ‚Ja', wie häufig?	___________
☐ Eigentumsdelikt (z. B. Diebstahl, Betrug)	Wenn ‚Ja', wie häufig?	___________
☐ Brandstiftung	Wenn ‚Ja', wie häufig?	___________
☐ Verkehrsdelikt	Wenn ‚Ja', wie häufig?	___________
☐ Vergehen gg. das BtMG	Wenn ‚Ja', wie häufig?	___________
☐ Sonstiges ________________________	Wie häufig?	___________

☐ Nein, kein erneutes verurteiltes Delikt
☐ Unbekannt

48. Flucht oder Entweichungen in den letzten 12 Monaten der Maßregelvollzugsbehandlung

☐ Ausbruch (Flucht unter Überwindung baulich-technischer Hindernisse)
☐ Entweichung (nicht nur kurzfristiger Entzug aus der Aufsicht von Mitarbeiter*innen während eines Ausgangs)
☐ Nicht-Rückkehr aus selbstständigen Lockerungen (Polizei informiert)
☐ Entweichung aus Probewohnen/Langzeitbeurlaubung
☐ Nein
☐ Unbekannt

49. Suizidversuche in den letzten 12 Monaten der Maßregelvollzugsbehandlung

☐ Ja
☐ Nein
☐ Unbekannt

50. Selbstverletzendes Verhalten in den letzten 12 Monaten der Maßregelvollzugsbehandlung

☐ Ja
☐ Nein
☐ Unbekannt

51. Aktuelle Lockerung

☐ Keine
☐ Begleiteter Ausgang mit Personal (auf geschlossenem Klinikgelände)
☐ Unbegleiteter Ausgang (auf geschlossenem Klinikgelände, ohne Personal)
☐ Begleiteter Ausgang mit Personal (außerhalb des geschlossenen Klinikgeländes)
☐ Unbegleiteter Ausgang (außerhalb des geschlossenen Klinikgeländes, ohne Personal)
☐ Belastungsbeurlaubung (Ausgang mit Übernachtung)
☐ Probewohnen/Langzeitbeurlaubung,
Seit wann (Monat/Jahr)?: ___________
☐ Unbekannt

52. Interne prognostische Beurteilung der Entlassung

☐ Bewährungsentlassung wahrscheinlich innerhalb der nächsten 6 Monate
☐ Bewährungsentlassung wahrscheinlich zwischen 6 und 12 Monaten
☐ Bewährungsentlassung wahrscheinlich zwischen 12 und 24 Monaten
☐ Bewährungsentlassung wahrscheinlich zwischen 2 und 5 Jahren
☐ Bewährungsentlassung wahrscheinlich in über 5 Jahren
☐ Bewährungsentlassung auch nach mehr als 5 Jahren unwahrscheinlich

Basismodul aktualisieren (17 Fragen)

1. Alter am Stichtag [z.B. 31.12.2022]

____

☐ Unbekannt

2. Aktueller Beziehungsstatus

☐ Ledig
☐ Verheiratet / in eingetragener Partnerschaft
☐ In sonstiger fester Partnerschaft
☐ Verwitwet
☐ Geschieden
☐ Unbekannt

3. Hat der/die Patient*in die Personensorge oder Teile dieser für ein oder mehrere Kinder?

☐ Ja
☐ Nein
☐ Unbekannt

4. Schulbildung am Stichtag [z.B. 31.12.2022]

☐ Förderschule
☐ Hauptschule
☐ Realschule/POS
☐ Abitur/Fachabitur
☐ Keiner
☐ Unbekannt

5. Berufsausbildung am Stichtag [z.B. 31.12.2022]

☐ Ungelernt/abgebrochene Berufsausbildung
☐ Abgeschlossene Berufsausbildung
☐ Abgeschlossenes Studium (FH/Universität)
☐ Keine
☐ Unbekannt

6. Aktuelle psychiatrische Diagnosen

Diagnose	Ja	ICD-10-Kode
F00-F09 Organische, einschließlich symptomatischer psychischer Störungen	☐	☐ F00. Demenz bei Alzheimer-Krankheit<br>☐ F01. Vaskuläre Demenz<br>☐ F02. Demenz bei anderenorts klassifizierten Krankheiten<br>☐ F03. Nicht näher bezeichnete Demenz

		☐ F04. Organisches amnestisches Syndrom, nicht durch Alkohol oder andere psychotrope Substanzen bedingt<br>☐ F05. Delir, nicht durch Alkohol oder andere psychotrope Substanzen bedingt<br>☐ F06. Andere psychische Störungen aufgrund einer Schädigung oder Funktionsstörung des Gehirns oder einer körperlichen Krankheit<br>☐ F07. Persönlichkeits- und Verhaltensstörung aufgrund einer Krankheit, Schädigung oder Funktionsstörung des Gehirns<br>☐ F09. Nicht näher bezeichnete organische oder symptomatische psychische Störung
F10-F19 Psychische und Verhaltensstörungen durch psychotrope Substanzen	☐	☐ F10. Psychische und Verhaltensstörungen durch Alkohol<br>☐ F11. Psychische und Verhaltensstörungen durch Opioide<br>☐ F12. Psychische und Verhaltensstörungen durch Cannabinoide<br>☐ F13. Psychische und Verhaltensstörungen durch Sedativa oder Hypnotika<br>☐ F14. Psychische und Verhaltensstörungen durch Kokain<br>☐ F15. Psychische und Verhaltensstörungen durch andere Stimulanzien, einschließlich Koffein<br>☐ F16. Psychische und Verhaltensstörungen durch Halluzinogene<br>☐ F17. Psychische und Verhaltensstörungen durch Tabak<br>☐ F18. Psychische und Verhaltensstörungen durch flüchtige Lösungsmittel<br>☐ F19. Psychische und Verhaltensstörungen durch multiplen Substanzgebrauch und Konsum anderer psychotroper Substanzen
F20-F29 Schizophrenie, schizotype und wahnhafte Störungen	☐	☐ F20. Schizophrenie<br>☐ F21. Schizotype Störung<br>☐ F22. Anhaltende wahnhafte Störungen<br>☐ F23. Akute vorübergehende psychotische Störungen<br>☐ F24. Induzierte wahnhafte Störung<br>☐ F25. Schizoaffektive Störungen<br>☐ F28. Sonstige nichtorganische psychotische Störungen<br>☐ F29. Nicht näher bezeichnete nichtorganische Psychose

F30-F39 Affektive Störungen	☐	☐ F30. Manische Episode<br>☐ F31. Bipolare affektive Störung<br>☐ F32. Depressive Episode<br>☐ F33. Rezidivierende depressive Störung<br>☐ F34. Anhaltende affektive Störungen<br>☐ F38. Andere affektive Störungen<br>☐ F39. Nicht näher bezeichnete affektive Störung
F40-F48 Neurotische, Belastungs- und somatoforme Störungen	☐	☐ F40. Phobische Störungen<br>☐ F41. Andere Angststörungen<br>☐ F42. Zwangsstörung<br>☐ F43. Reaktionen auf schwere Belastungen und Anpassungsstörungen<br>☐ F44. Dissoziative Störungen [Konversionsstörungen]<br>☐ F45. Somatoforme Störungen<br>☐ F48. Andere neurotische Störungen
F50-F59 Verhaltensauffälligkeiten mit körperlichen Störungen und Faktoren	☐	☐ F50. Essstörungen<br>☐ F51. Nichtorganische Schlafstörungen<br>☐ F52. Sexuelle Funktionsstörungen, nicht verursacht durch eine organische Störung oder Krankheit<br>☐ F53. Psychische oder Verhaltensstörungen im Wochenbett, anderenorts nicht klassifiziert<br>☐ F54. Psychologische Faktoren oder Verhaltensfaktoren bei anderenorts klassifizierten Krankheiten<br>☐ F55. Schädlicher Gebrauch von nichtabhängigkeitserzeugenden Substanzen<br>☐ F59. Nicht näher bezeichnete Verhaltensauffälligkeiten bei körperlichen Störungen und Faktoren
F60-F69 Persönlichkeits- und Verhaltensstörungen	☐	☐ F60. Spezifische Persönlichkeitsstörung<br>☐ F60.0. Paranoide Persönlichkeitsstörung<br>☐ F60.1. Schizoide Persönlichkeitsstörung<br>☐ F60.2. Dissoziale Persönlichkeitsstörung<br>☐ F60.3. Emotional instabile Persönlichkeitsstörung<br>☐ F60.4. Histrionische Persönlichkeitsstörung<br>☐ F60.5. Anankastische [zwanghafte] Persönlichkeitsstörung<br>☐ F60.6. Ängstliche (vermeidende) Persönlichkeitsstörung<br>☐ F60.7. Abhängige (asthenische) Persönlichkeitsstörung<br>☐ F60.8. Sonstige spezifische Persönlichkeitsstörungen

		☐ F60.9. Persönlichkeitsstörung, nicht näher bezeichnet<br>☐ F61. Kombinierte und andere Persönlichkeitsstörungen<br>☐ F62. Andauernde Persönlichkeitsänderungen, nicht Folge einer Schädigung oder Krankheit des Gehirns<br>☐ F63. Abnorme Gewohnheiten und Störungen der Impulskontrolle<br>☐ F64. Störungen der Geschlechtsidentität<br>☐ F65. Störungen der Sexualpräferenz<br>☐ F66. Psychische und Verhaltensstörungen in Verbindung mit der sexuellen Entwicklung und Orientierung<br>☐ F68. Andere Persönlichkeits- und Verhaltensstörungen<br>☐ F69. Nicht näher bezeichnete Persönlichkeits- und Verhaltensstörung
F70-F79 Intelligenzminderung	☐	☐ F70. Leichte Intelligenzminderung<br>☐ F71. Mittelgradige Intelligenzminderung<br>☐ F72. Schwere Intelligenzminderung<br>☐ F73. Schwerste Intelligenzminderung<br>☐ F74. Dissoziierte Intelligenz<br>☐ F78. Andere Intelligenzminderung<br>☐ F79. Nicht näher bezeichnete Intelligenzminderung
F80-F89 Entwicklungsstörungen	☐	☐ F80. Umschriebene Entwicklungsstörungen des Sprechens und der Sprache<br>☐ F81. Umschriebene Entwicklungsstörungen schulischer Fertigkeiten<br>☐ F82. Umschriebene Entwicklungsstörung der motorischen Funktionen<br>☐ F83. Kombinierte umschriebene Entwicklungsstörungen<br>☐ F84. Tief greifende Entwicklungsstörungen<br>☐ F88. Andere Entwicklungsstörungen<br>☐ F89. Nicht näher bezeichnete Entwicklungsstörung
F90-F98 Verhaltens- und emotionale Störungen mit Beginn in der Kindheit und Jugend	☐	☐ F90. Hyperkinetische Störungen<br>☐ F91. Störungen des Sozialverhaltens<br>☐ F92. Kombinierte Störung des Sozialverhaltens und der Emotionen<br>☐ F93. Emotionale Störungen des Kindesalters<br>☐ F94. Störungen sozialer Funktionen mit Beginn in der Kindheit und Jugend<br>☐ F95. Ticstörungen<br>☐ F98. Andere Verhaltens- und emotionale Störungen mit Beginn in der Kindheit und Jugend

F99-F99 Nicht näher bezeichnete psychische Störungen	☐	☐ F99. Psychische Störung ohne nähere Angabe

7. Falls psychische und Verhaltensstörungen durch psychotrope Substanzen (F10-F19), welches ist/sind Hauptkonsummittel?

(Mehrfachnennung möglich, bis zu drei Nennungen)

☐ Alkohol
☐ Opioide
☐ Cannabinoide
☐ Sedativa oder Hypnotika
☐ Kokain
☐ Andere Stimulanzien
☐ Halluzinogene
☐ Flüchtige Lösungsmittel
☐ Medikamente
☐ Amphetamine
☐ NPS (Neue psychoaktive Substanzen)
☐ Sonstiges ___________________________
☐ Unbekannt

8. Aktuelle somatische Diagnosen

(Mehrfachnennung möglich)

☐ Bluthochdruck
☐ Diabetes
☐ Sonstige Herz-Kreislauf-Erkrankungen
☐ Adipositas (BMI> 30)
☐ Krebs
☐ Epilepsie
☐ Erkrankung der Atemwege
bitte angeben _________________________
☐ Erkrankung des Magen-Darm-Systems
bitte angeben _________________________
☐ Erkrankung des Bewegungsapparates
bitte angeben _________________________
☐ Infektionserkrankungen (z.B. HIV, Hepatitis)
bitte angeben _________________________
☐ Andere relevante Erkrankungen
bitte angeben _________________________
☐ Keine
☐ Unbekannt

9. Raucher*in?

☐ Ja

☐ Nein
☐ Unbekannt

10. Art der derzeitigen Station

☐ Aufnahmestation
☐ Hochgesicherte Station
☐ Reguläre Behandlungsstation
☐ Long-Stay-Station
☐ Station zur Entlassungsvorbereitung
☐ Außenwohnstätte
☐ Langzeitbeurlaubung
☐ Sonstige ________________________
☐ Unbekannt

11. Vorfälle in den letzten 12 Monaten der Maßregelvollzugsbehandlung

Mehrfachnennung möglich. Fragen zu Flucht kommen später in der Umfrage.

a. Vorfälle in der Klinik

☐ Nein, keine

☐ verbale Aggression gegenüber Mitarbeiterinnen	Wenn ‚Ja', wie häufig?	__________
☐ körperliche Übergriffe gegenüber Mitarbeiterinnen	Wenn ‚Ja', wie häufig?	__________
☐ Sexuelle Übergriffe gegenüber Mitarbeiterinnen	Wenn ‚Ja', wie häufig?	__________
☐ verbale Aggression gegenüber Patienten*innen	Wenn ‚Ja', wie häufig?	__________
☐ körperliche Übergriffe gegenüber Patienten*innen	Wenn ‚Ja', wie häufig?	__________
☐ Sexuelle Übergriffe gegenüber Patienten*innen	Wenn ‚Ja', wie häufig?	__________
☐ Sachbeschädigung	Wenn ‚Ja', wie häufig?	__________
☐ Brandstiftung	Wenn ‚Ja', wie häufig?	__________
☐ Substanzkonsum	Wenn ‚Ja', wie häufig?	__________
☐ Scheitern Langzeitbeurlaubung/ Probewohnen	Wenn ‚Ja', wie häufig?	__________

☐ Sonstiges ______________________ Wie häufig? __________

☐ Unbekannt

b. Vorfälle während Ausgang (begleiteter oder unbegleiteter)

☐ Keine

☐ verbale Aggression gegenüber Mitarbeiter*innen	Wenn ‚Ja', wie häufig?	__________
☐ körperliche Übergriffe gegenüber Mitarbeiter*innen	Wenn ‚Ja', wie häufig?	__________
☐ Sexuelle Übergriffe gegenüber Mitarbeiter*innen	Wenn ‚Ja', wie häufig?	__________
☐ verbale Aggression gegenüber Patienten*innen	Wenn ‚Ja', wie häufig?	__________
☐ körperliche Übergriffe gegenüber Patienten*innen	Wenn ‚Ja', wie häufig?	__________
☐ Sexuelle Übergriffe gegenüber Patienten*innen	Wenn ‚Ja', wie häufig?	__________
☐ Sachbeschädigung	Wenn ‚Ja', wie häufig?	__________
☐ Brandstiftung	Wenn ‚Ja', wie häufig?	__________
☐ Substanzkonsum	Wenn ‚Ja', wie häufig?	__________
☐ Sonstiges ______________________	Wie häufig?	__________

☐ Unbekannt

c. Vorfälle während Langzeitbeurlaubung/Probewohnen

☐ Keine

☐ verbale Aggression gegenüber Mitarbeiter*innen	Wenn ‚Ja', wie häufig?	__________
☐ körperliche Übergriffe gegenüber Mitarbeiter*innen	Wenn ‚Ja', wie häufig?	__________

☐ Sexuelle Übergriffe gegenüber Mitarbeiter*innen	Wenn ‚Ja', wie häufig?	__________
☐ verbale Aggression gegenüber Patienten*innen	Wenn ‚Ja', wie häufig?	__________
☐ körperliche Übergriffe gegenüber Patienten*innen	Wenn ‚Ja', wie häufig?	__________
☐ Sexuelle Übergriffe gegenüber Patienten*innen	Wenn ‚Ja', wie häufig?	__________
☐ Sachbeschädigung	Wenn ‚Ja', wie häufig?	__________
☐ Brandstiftung	Wenn ‚Ja', wie häufig?	__________
☐ Substanzkonsum	Wenn ‚Ja', wie häufig?	__________
☐ Sonstiges ______________________	Wie häufig?	__________
☐ Unbekannt		

i. **Falls ‚ja', führte dies zu einer Wiederaufnahme in der Klinik?**

☐ Ja
☐ Nein
☐ Unbekannt

12. Strafrechtliche Verurteilungen für im Maßregelvollzug begangene Straftaten in den letzten 12 Monaten

(Mehrfachnennung möglich; Tatbegehung in Klinik, Ausgang und Langzeitbeurlaubung/Probewohnen)

☐ Tötungsdelikt	Wenn ‚Ja', wie häufig?	__________
☐ Versuchtes Tötungsdelikt	Wenn ‚Ja', wie häufig?	__________
☐ Sonstiges Körperverletzungsdelikt	Wenn ‚Ja', wie häufig?	__________
☐ Sexualdelikt gegen Erwachsene	Wenn ‚Ja', wie häufig?	__________
☐ Sexualdelikt gegen Kinder	Wenn ‚Ja', wie häufig?	__________
☐ Sonstiges Gewaltdelikt (z.B. Raub, Erpressung, Nötigung, Bedrohung)	Wenn ‚Ja', wie häufig?	__________
☐ Eigentumsdelikt (z. B. Diebstahl, Betrug)	Wenn ‚Ja', wie häufig?	__________

☐ Brandstiftung — Wenn ‚Ja‘, wie häufig? __________

☐ Verkehrsdelikt — Wenn ‚Ja‘, wie häufig? __________

☐ Vergehen gg. das BtMG — Wenn ‚Ja‘, wie häufig? __________

☐ Sonstiges ______________________ — Wie häufig? __________

☐ Nein, kein erneutes verurteiltes Delikt
☐ Unbekannt

13. Flucht oder Entweichungen in den letzten 12 Monaten der Maßregelvollzugsbehandlung

☐ Ausbruch (Flucht unter Überwindung baulich-technischer Hindernisse)
☐ Entweichung (nicht nur kurzfristiger Entzug aus der Aufsicht von Mitarbeiter*innen während eines Ausgangs)
☐ Nicht-Rückkehr aus selbstständigen Lockerungen (Polizei informiert)
☐ Entweichung aus Probewohnen/Langzeitbeurlaubung
☐ Nein
☐ Unbekannt

14. Suizidversuche in den letzten 12 Monaten der Maßregelvollzugsbehandlung

☐ Ja
☐ Nein
☐ Unbekannt

15. Selbstverletzendes Verhalten in den letzten 12 Monaten der Maßregelvollzugsbehandlung

☐ Ja
☐ Nein
☐ Unbekannt

16. Aktuelle Lockerung

☐ Keine
☐ Begleiteter Ausgang mit Personal (auf geschlossenem Klinikgelände)
☐ Unbegleiteter Ausgang (auf geschlossenem Klinikgelände, ohne Personal)
☐ Begleiteter Ausgang mit Personal (außerhalb des geschlossenen Klinikgeländes)
☐ Unbegleiteter Ausgang (außerhalb des geschlossenen Klinikgeländes, ohne Personal)
☐ Belastungsbeurlaubung (Ausgang mit Übernachtung)
☐ Probewohnen/Langzeitbeurlaubung,
Seit wann (Monate/Jahr): ___________
☐ Unbekannt

17. Interne prognostische Beurteilung der Entlassung

☐ Bewährungsentlassung wahrscheinlich innerhalb der nächsten 6 Monate
☐ Bewährungsentlassung wahrscheinlich zwischen 6 und 12 Monaten
☐ Bewährungsentlassung wahrscheinlich zwischen 12 und 24 Monaten
☐ Bewährungsentlassung wahrscheinlich zwischen 2 und 5 Jahren
☐ Bewährungsentlassung wahrscheinlich in über 5 Jahren
☐ Bewährungsentlassung auch nach mehr als 5 Jahren unwahrscheinlich

Klinisches Forschungsmodul (11 Fragen)

1. Durchschnittliche Anzahl von privaten Besuchen in den letzten drei Monaten:

☐ Weniger als einmal im Monat
☐ 1-2 mal pro Monat
☐ 3-5 mal pro Monat
☐ >5 mal pro Monat
☐ Kein Besuch
☐ Unbekannt

2. Behandlungsprogramme in den letzten 12 Monaten der Maßregelvollzugsbehandlung

Innerhalb der letzten **12** Monate (oder seit Aufnahme)
(Mehrfachnennung möglich)

Komplexe Interventionen

Behandlungsprogramm	Beteiligung
Dialektisch-Behaviorale Therapie (DBT, alle Unterformen)	☐ Nimmt derzeit teil<br>☐ Regulärer Therapieabschluss<br>☐ Irreguläres Therapieende
Sexualstrattäterprogramm	☐ Nimmt derzeit teil<br>☐ Regulärer Therapieabschluss<br>☐ Irreguläres Therapieende
Gewaltstraftäterprogramm	☐ Nimmt derzeit teil<br>☐ Regulärer Therapieabschluss<br>☐ Irreguläres Therapieende

Gruppentherapie/-programm

Behandlungsprogramm	Beteiligung
R & R	☐ Nimmt derzeit teil<br>☐ Regulärer Therapieabschluss<br>☐ Irreguläres Therapieende

Schematherapie	☐ Nimmt derzeit teil<br>☐ Regulärer Therapieabschluss<br>☐ Irreguläres Therapieende
Sonstige verhaltenstherapeutisch orientierte Gruppe	☐ Nimmt derzeit teil<br>☐ Regulärer Therapieabschluss<br>☐ Irreguläres Therapieende
Psychodynamisch/-analytisch orientierte Gruppe	☐ Nimmt derzeit teil<br>☐ Regulärer Therapieabschluss<br>☐ Irreguläres Therapieende
Psychoedukation	☐ Nimmt derzeit teil<br>☐ Regulärer Therapieabschluss<br>☐ Irreguläres Therapieende
Soziales /Emotionales Kompetenztraining	☐ Nimmt derzeit teil<br>☐ Regulärer Therapieabschluss<br>☐ Irreguläres Therapieende
Musiktherapie	☐ Nimmt derzeit teil<br>☐ Regulärer Therapieabschluss<br>☐ Irreguläres Therapieende
Kunsttherapie	☐ Nimmt derzeit teil<br>☐ Regulärer Therapieabschluss<br>☐ Irreguläres Therapieende

<u>Einzeltherapie</u>

**Orientierung**	**Beteiligung**

Verhaltenstherapeutisch	☐ Nimmt derzeit teil<br>☐ Regulärer Therapieabschluss<br>☐ Irreguläres Therapieende
Schematherapeutisch	☐ Nimmt derzeit teil<br>☐ Regulärer Therapieabschluss<br>☐ Irreguläres Therapieende
Psychodynamisch/-analytisch	☐ Nimmt derzeit teil<br>☐ Regulärer Therapieabschluss<br>☐ Irreguläres Therapieende
Familientherapie	☐ Nimmt derzeit teil<br>☐ Regulärer Therapieabschluss<br>☐ Irreguläres Therapieende
Musiktherapie	☐ Nimmt derzeit teil<br>☐ Regulärer Therapieabschluss<br>☐ Irregulärer Therapieabschluss
Kunsttherapie	☐ Nimmt derzeit teil<br>☐ Regulärer Therapieabschluss<br>☐ Irregulärer Therapieabschluss
Eklektisch / Sonstige Orientierung	☐ Nimmt derzeit teil<br>☐ Regulärer Therapieabschluss<br>☐ Irregulärer Therapieabschluss

3. Durchschnittliche Stundenzahl Komplementärtherapien / Arbeit pro Woche in den letzten 3 Monaten der Maßregelvollzugsbehandlung

Innerhalb der letzten **drei** Monate (oder seit der Aufnahme)
Mehrfachnennung möglich

**Behandlungsprogramme**	Durchschnittliche Stundenzahl pro Woche
Ergotherapie	☐ 0

	☐ 1-2<br>☐ 3-5<br>☐ 6-10<br>☐ 11-15<br>☐ 16-20<br>☐ 21+<br>☐ Unbekannt
Externes Praktikum/Arbeitsplatz	☐ 0<br>☐ 1-2<br>☐ 3-5<br>☐ 6-10<br>☐ 11-15<br>☐ 16-20<br>☐ 21+<br>☐ Unbekannt
Sporttherapie	☐ 0<br>☐ 1-2<br>☐ 3-5<br>☐ 6-10<br>☐ >10<br>☐ Unbekannt

4. Berufsqualifizierender Abschluss innerhalb der letzten 12 Monate der Maßregelvollzugsbehandlung

Innerhalb der letzten **12** Monate (oder seit der Aufnahme)
Mehrfachnennung möglich

Abschluss	Beteiligung	Welcher Typ / Abschluss?
Besucht Schulkurs (z. B. Realschulkurs)	☐ Nimmt derzeit teil<br>☐ Teilgenommen, abgeschlossen<br>☐ Teilgenommen, nicht abgeschlossen	____________<br>____________<br>____________
Sonstiger Unterricht (z. B. Sprachunterricht, Förderunterricht)	☐ Nimmt derzeit teil<br>☐ Teilgenommen, abgeschlossen<br>☐ Teilgenommen, nicht abgeschlossen	____________<br>____________<br>____________
Berufsausbildung	☐ Nimmt derzeit teil<br>☐ Teilgenommen, abgeschlossen	____________<br>____________

	☐ Teilgenommen, nicht abgeschlossen	______________
Hochschulabschluss	☐ Nimmt derzeit teil<br>☐ Teilgenommen, abgeschlossen<br>☐ Teilgenommen, nicht abgeschlossen	______________<br>______________<br>______________
Sonstige Qualifikationen (z. B. Gabelstaplerschein, Schweißerschein)	☐ Nimmt derzeit teil<br>☐ Teilgenommen, abgeschlossen<br>☐ Teilgenommen, nicht abgeschlossen	______________<br>______________<br>______________
Keine	☐	
Unbekannt	☐	

5. Teilnahme an Freizeitaktivitäten in den letzten 3 Monaten der Maßregelvollzugsbehandlung (innerhalb oder außerhalb der Klinik)

(z.B. Sport, Fitnessstudio, Hobbys, Vereins- oder Teamaktivitäten)
Innerhalb der letzten **drei** Monate (oder seit der Aufnahme)

Freizeitaktivität: ______________	☐ weniger als einmal im Monat<br>☐ 1-2<br>☐ 3-5<br>☐ 5-9<br>☐ 10+
Freizeitaktivität: ______________	☐ weniger als einmal im Monat<br>☐ 1-2<br>☐ 3-5<br>☐ 5-9<br>☐ 10+
Freizeitaktivität: ______________	☐ weniger als einmal im Monat<br>☐ 1-2<br>☐ 3-5<br>☐ 5-9<br>☐ 10+
Keine Freizeitaktivitäten	☐
Unbekannt	☐

6. Derzeitige regelmäßige psychotrope Medikation

(Regelmäßig mindestens über einen Zeitraum von **drei** Monaten. Mehrfachnennungen möglich.)

☐ Typische Antipsychotika – oral
☐ Atypische Antipsychotika – oral (außer Clozapin)
☐ Typische Antipsychotika – Depot
☐ Atypische Antipsychotika – Depot
☐ Clozapin
☐ Antidepressiva
☐ Benzodiazepine
☐ Hypnotika
☐ Stimmungsstabilisierende Medikamente, inkl. Antiepileptika
☐ Medikamente zur Behandlung von AHDS
☐ antilibidinale Medikation
☐ Antiparkinsonmittel
☐ Substitutionstherapie
☐ Andere, benennen
☐ Keine
☐ Unbekannt

7. Zwangsmedikation in den letzten 3 Monaten der Maßregelvollzugsbehandlung

☐ Ja (Einmal oder mehrmals)
☐ Keine
☐ Unbekannt

8. Bitte geben Sie hier Ihre fachliche Einschätzung an.

	Stimme gar nicht zu stimme voll zu					Kann ich nicht beurteilen
	0	1	2	3	4	
(a) Der/die Patient*in nimmt zuverlässig am Behandlungsprogramm teil.	☐	☐	☐	☐	☐	☐
(b) Der/die Patient*in übernimmt für sein/ihr Tathandeln Verantwortung.	☐	☐	☐	☐	☐	☐
(c) Der/die Patient*in konnte in der Therapie Opferempathie entwickeln.	☐	☐	☐	☐	☐	☐
(d) Der/die Patient*in zeigt Einsicht in die Störung.	☐	☐	☐	☐	☐	☐
(e) Der/die Patient*in setzt sich aktiv mit seinen/ihren Delikten auseinander und	☐	☐	☐	☐	☐	☐

hat ein Verständnis wesentlicher Risikofaktoren entwickelt.						
(f) Der/die Patient*in ist impulsiv.	☐	☐	☐	☐	☐	☐
(g) Der/die Patient*in zeigt Motivation zur Behandlung.	☐	☐	☐	☐	☐	☐
(h) Der/die Patient*in handelt proaktiv, um persönliche Probleme zu lösen.	☐	☐	☐	☐	☐	☐
(i) Der/die Patient*in nimmt alle verschriebenen Medikamente zuverlässig ein.	☐	☐	☐	☐	☐	☐

9. Fixierung in den letzten 3 Monaten der Maßregelvollzugsbehandlung

Gesamtzahl in den letzten drei Monaten

☐ 0
☐ 1-2
☐ 3-5
☐ 6-10
☐ >10
☐ Unbekannt

10. Isolierung / Krisenraumunterbringung in den letzten 3 Monaten der Maßregelvollzugsbehandlung

Gesamtzahl in den letzten drei Monaten

☐ 0
☐ 1-2
☐ 3-5
☐ 6-10
☐ >10
☐ Unbekannt

11. Wurden standardisierte Instrumente angewandt?

Bitte geben Sie das Testergebnis (numerisch oder summativ z.B. niedrig, hoch entsprechend der Einstufung des Fragebogens) für die verwendeten Instrumente ein. Mehrfachnennungen möglich. Ignorieren, wenn nicht relevant.

a. Risiko

HCR-20
Datum des letzten Ergebnisses: ________________
Version: _______

H: _______
C: _______
R: _______

Gesamt: _______

Level of Service Inventory-Revised (LSI-R)
Datum des letzten Ergebnisses: _________________

Kriminelle Vorgeschichte: _______
Ausbildung/Erwerbstätigkeit: _______
Finanzielle Situation: _______
Familie/Partnerschaft: _______
Wohnsituation: _______
Freizeitgestaltung: _______
Freundschaften/Bekanntschaften: _______
Alkohol-/Drogenproblematik: _______
Emotionale Beeinträchtigung: _______
Einstellungen/Orientierungen/Werte: _______

Gesamt: _______

Violence Risk Appraisal Guides (VRAG)
Datum des letzten Ergebnisses: _________________

Gesamt: _______

STATIC-99
Datum des letzten Ergebnisses: _________________

Gesamt: _______

Sexual Offender Risk Appraisal Guide (SORAG)
Datum des letzten Ergebnisses: _________________

Gesamt: _______

---

b. Funktionsniveaus

Global Assessment of Functioning (GAF)
Datum des letzten Ergebnisses: _________________

Psychisches Funktionsniveau: _______
Soziales Funktionsniveau: _______

Berufliches Funktionsniveau: _______

Gesamt: _______

Skala zur Erfassung des sozialen und beruflichen Funktionsniveaus (SOFAS)
Datum des letzten Ergebnisses:_________________

Gesamt: _______

Barratt Impulsiveness Scale (BIS)
Datum des letzten Ergebnisses:_________________

Aufmerksamkeit: _______
Kognitive Flexibilität: _______
Motorische Impulsivität: _______
Beharrlichkeit: _______
Selbstkontrolle: _______
Kognitive Instabilität: _______

Gesamt: _______

Recovery Assessment Scale (RAS; Nutzende- und Fachpersonen-Version)
Datum des letzten Ergebnisses:_________________

Lebensziele: _______
Beteiligung: _______
Vielfalt der Behandlungsoptionen: _______
Wahlmöglichkeiten: _______
Individuell abgestimmte Dienste: _______

Gesamt: _______

Essen Climate Evaluation Schema (EssenCES, Patientenversion)
Datum des letzten Ergebnisses:_________________

Zusammenhalt der Patienten: _______
Sicherheitserleben: _______
Therapeutischer Halt: _______

Gesamt: _______

---

c. Symptome

Positive and Negative Symptom Scale (PANSS)
Datum des letzten Ergebnisses:_________________

Positiv Skala: _______
Negativ Skala: _______
Generelle Psychopathologische: _______

Gesamt: _______

Brief Psychiatric Rating Scale (BPRS)
Datum des letzten Ergebnisses:_________________

Gesamt: _______

Beck Depressions-Inventar (BDI)
Datum des letzten Ergebnisses:_________________
Version: _______

Gesamt: _______

Beck Anxiety Inventory (BAI)
Datum des letzten Ergebnisses:_________________

Gesamt: _______

Hare Psychopathy-Checklist (PCL-R)
Datum des letzten Ergebnisses:_________________

Faktor 1: ________
„einen selbstsüchtigen, gemütsarmen Menschen [charakterisiert], der gewissenlos andere gebraucht“

Faktor 2: ________
„einen chronisch instabilen und antisozialen Lebensstil und sozial abweichendes Verhalten“

Gesamt: ________

Sonstiges Instrument (bitte Instrument, Ergebnis und Testdatum angeben)

Instrument	Ergebnis	Datum des letzten Ergebnisses
______________	______________	______________

Entlassungsmodul (8 Fragen)

1. Verlegungsdatum/Entlassungsdatum

z.B.: 01.01.1924

____

2. Entlassgrundlage

☐ Aussetzen MRV-Unterbringung auf Bewährung
☐ Verhältnismäßigkeit
☐ Andere _____________________
☐ Unbekannt

3. Wohin wurde der/die Patient*in verlegt?

☐ Wohnen mit Partner*in
☐ Wohnen bei Angehörigen (z.B. Geschwister, Eltern, Pflegeeltern)
☐ Betreutes Wohnen
☐ Wohnheim (ink. Nachsorgeeinrichtung, Übergangswohnheim)
☐ Pflegeheim
☐ Andere forensische Klinik
☐ Justizvollzugsanstalt
☐ Sonstiges
☐ Unbekannt

4. Sozialkontakte bei Entlassung

Mehrfachnennung möglich
☐ Feste Partnerschaft
☐ Kinder
☐ Sonstige Familie
☐ Freunde
☐ Andere __________________________
☐ Kein
☐ Unbekannt

5. Ist derzeit erkennbar, dass der/die Patient*in nach seiner/ihrer Entlassung wahrscheinlich einer Beschäftigung oder Ausbildung nachgeht?

☐ Ja
Wenn bekannt, was: __________________________
☐ Mögliche Beschäftigung oder Ausbildung
☐ Kein
☐ Unbekannt

6. Ambulant psychiatrische Weiterbehandlung geplant

☐ Allgemeine
☐ Forensische
☐ Andere: ___________________
☐ Keine
☐ Unbekannt

7. Führungsaufsicht?

☐ Ja
Wenn ‚Ja' Dauer: ___________________________
☐ Nein
☐ Unbekannt

8. Weisungen nach § 68b (1) StGB

☐ Den Wohn- oder Aufenthaltsort oder einen bestimmten Bereich nicht ohne Erlaubnis zu verlassen
☐ sich nicht anbestimmter Orten aufzuhalten
☐ zu bestimmten Personen / Gruppen keinen Kontakt aufzunehmen
☐ bestimmte Tätigkeiten nicht auszuüben
☐ bestimmte Gegenstände nicht zu besitzen
☐ bestimmte Fahrzeuge nicht zu halten oder zu führen
☐ sich zu bestimmten Zeiten bei der Aufsichtsstelle zu melden
☐ den Wechsel von Wohnung oder Arbeitsplatz zu melden
☐ sich bei Erwerbslosigkeit ber der zuständigen Agentur für Arbeit zu melden
☐ keine alkoholischen Getränke oder andere berauschende Mittel zu sich zu nehmen
☐ sich bei einer Ärzt*in, Psychotherapeutin*en oder einer forensischen Ambulanz vorzustellen
☐ die elektronische Überwachung zuzulassen
☐ Andere: ___________________________
☐ Keine
☐ Unbekannt
